# A randomized trial evaluating the safety profile of sugammadex in high surgical risk ASA physical class 3 or 4 participants

**DOI:** 10.1186/s12871-021-01477-5

**Published:** 2021-10-28

**Authors:** W. Joseph Herring, Yuki Mukai, Aobo Wang, Jeannine Lutkiewicz, John F. Lombard, Li Lin, Molly Watkins, David M. Broussard, Manfred Blobner

**Affiliations:** 1 Department of Clinical Research, Merck & Co., Inc., Kenilworth, NJ USA; 2 Ochsner Clinic Foundation, New Orleans, LA USA; 3 Department of Anesthesiology and Intensive Care Medicine, School of Medicine, Technical University of Munich, Munich, Germany; 4 Department of Anesthesiology and Intensive Care Medicine, Faculty of Medicine, University of Ulm, Ulm, Germany

**Keywords:** Sugammadex: safety, ASA physical class 3 or 4

## Abstract

**Background:**

The aim of this randomized, double-blind trial was to evaluate the safety and tolerability profile, including cardiac safety, of sugammadex-mediated recovery from neuromuscular block in participants undergoing surgery who met the American Society of Anesthesiologists (ASA) Physical Class 3 or 4 criteria. Specifically, this study assessed the impact of sugammadex on cardiac adverse events (AEs) and other prespecified AEs of clinical interest.

**Methods:**

Participants meeting ASA Class 3 and 4 criteria were stratified by ASA Class and NMBA (rocuronium or vecuronium) then randomized to one of the following: 1) Moderate neuromuscular block, sugammadex 2 mg/kg; 2) Moderate neuromuscular block, neostigmine and glycopyrrolate (neostigmine/glycopyrrolate); 3) Deep neuromuscular block, sugammadex 4 mg/kg; 4) Deep neuromuscular block, sugammadex 16 mg/kg (rocuronium only). Primary endpoints included incidences of treatment-emergent (TE) sinus bradycardia, TE sinus tachycardia and other TE cardiac arrhythmias.

**Results:**

Of 344 participants randomized, 331 received treatment (61% male, BMI 28.5 ± 5.3 kg/m^2^, age 69 ± 11 years). Incidence of TE sinus bradycardia was significantly lower in the sugammadex 2 mg/kg group vs neostigmine/glycopyrrolate. The incidence of TE sinus tachycardia was significantly lower in the sugammadex 2 and 4 mg/kg groups vs neostigmine/glycopyrrolate. No significant differences in other TE cardiac arrythmias were seen between sugammadex groups and neostigmine/glycopyrrolate. There were no cases of adjudicated anaphylaxis or hypersensitivity reactions in this study.

**Conclusions:**

Compared with neostigmine/glycopyrrolate, incidence of TE sinus bradycardia was significantly lower with sugammadex 2 mg/kg and incidence of TE sinus tachycardia was significantly lower with sugammadex 2 mg/kg and 4 mg/kg. These results support the safety of sugammadex for reversing rocuronium- or vecuronium-induced moderate and deep neuromuscular block in ASA Class 3 or 4 participants.

**Trial registration:**

ClinicalTrials.gov Identifier: NCT03346057.

**Supplementary Information:**

The online version contains supplementary material available at 10.1186/s12871-021-01477-5.

## Background

Sugammadex (Bridion®, Merck & Co., Inc., Kenilworth, NJ, USA), a modified cyclodextrin, reverses neuromuscular blockade from the neuromuscular blocking agents, rocuronium and vecuronium [1, 2]. Sugammadex encapsulates unbound rocuronium or vecuronium providing rapid and predictable reversal, and avoiding anticholinesterase side effects and antimuscarinic drug use [3–5]. Studies confirm the safety and efficacy of sugammadex for reversal of moderate or deep, rocuronium- or vecuronium-induced neuromuscular block [6–11]; however, randomized clinical trial data are limited in higher surgical risk ASA Physical Class 3 (defined as severe disease) or 4 (defined as severe systemic disease that is a constant threat to life) participants [2, 6].

Given the uniqueness of its engineered mechanism of action, sugammadex binds to no known human receptors and has no intrinsic biological activity, consistent with the notion that it is effectively inert. Results of in vitro, preclinical, and dedicated human studies have indicated that sugammadex has no direct effect on heart rate or electrical conduction within the heart [3, 12–16]. The sugammadex mechanism of action does not suggest any effect on autonomic tone, cardiac impulse generation or cardiac conduction. In the overall comprehensive evaluation of cardiac safety in the sugammadex development program, bradycardia was infrequently observed [16–20]. Notably, bradycardia was not detected in the population of healthy participants studied who received only sugammadex without neuromuscular blocking agent (NMBA) [12, 13]. In clinical studies involving surgical patients, the rates of bradycardia observed with sugammadex administration consistently appeared lower than that of comparator neostigmine, a reversal agent for which co-administered countermeasures against bradycardia are typically given [16].

Nevertheless, available approved clinical reports suggest that in rare cases, neuromuscular block reversal with sugammadex may be associated with marked bradycardia [2, 21]. This risk of bradycardia, which is readily detectable in the perioperative setting, typically responds well to usual intervention and is appropriately addressed through existing product labeling [17]. While evidence is lacking to suggest a direct causal effect of sugammadex on heart rate, the clinical pattern of bradycardia does not rule out the possibility of an undefined indirect relationship.

Because ASA Physical Class 3 and 4 surgical patients may, by definition, be at higher risk for safety events including arrhythmias, this study was conducted to evaluate the overall safety profile of sugammadex in this important subpopulation with a focus on the comparative incidence of treatment-emergent (TE) cardiac arrhythmias after sugammadex vs neostigmine/glycopyrrolate administration [22]. The primary safety endpoints included incidences of TE sinus bradycardia, TE sinus tachycardia and other TE cardiac arrhythmias. Events of clinical interest (ECI) included clinically relevant (CR) sinus bradycardia, CR sinus tachycardia, other CR cardiac arrhythmias, drug-induced liver injury, and adjudicated hypersensitivity and anaphylaxis.

## Methods

Institutional review board committees at each site approved this randomized, active comparator-controlled, multi-site, parallel-group, double-blind safety study, conducted at 27 sites in 4 countries from December 2017 to September 2019. This study was registered on clinicaltrials.gov registry on 17/11/2017 (Study protocol 145; Clinicaltrials.gov: NCT03346057). All participants provided written, informed consent. Study protocol is provided in Supplementary Information (Additional file 1).

The physician investigators at all US sites were board- certified anesthesiologists by the American Board of Anesthesiology or certified to practice anesthesiology in the United States [23]. Participating investigators within the European Union were licensed physicians with specialties in anesthesiology in their respective countries, requiring comprehensive training meeting and/or exceeding requirements in the United States [23]. All participating investigators met Health Authority qualifications to serve as investigators in clinical trials [23]. The study was conducted in accordance with principles of Good Clinical Practice and followed the recommendations of CONSORT guidelines (Additional file 2). The following independent ethics committees were: Ethik Kommission Der Stadt Wien (Austria) for 3 sites (Sozialmedizinisches Zentrum Ost Donauspital, A.O. Krankenhaus Dornbirn, and Landeskrankenhaus Feldkirch), De Videnskabsetiske Komiteer for Region Hovedstaden (Denmark) for 4 sites (Bispebjerg og Frederiksberg Hospital, Aarhus Universitets Hospital, Rigshospitalet- The Juliane Marie Centre, and Regionshospitalet Viborg), Ethik-Kommission bei der Landesaertzekammer Baden-Württemberg (Germany), University of California Davis Medical Center Institutional Review Board (US), Western Institutional Review Board (US) for 7 sites (Temple University Hospital, Jackson Memorial Hospital, Saint Peter’s University Hospital, University Banner Medical Center, Beaumont Hospital -Royal Oak, University of Alabama -Birmingham, and Jersey Shore University Medical Center), Ochsner Clinic Foundation Institutional Review Board (US), Zablocki VA Medical Center Institutional Review Board (US), Mission Health (US), Copernicus Group Independent Review Board (US) for 2 sites (Tulane University and Hermann Drive Surgical Center), University of Missouri – Columbia Institutional Review Board (US), Loma Linda University Health Institutional Review Board (US), Cleveland Clinic Institutional Review Board (US), Vanderbilt Human Research Protection Program (US), Partners Human Research Committee (US), and University of Kansas Medical Center Institutional Review Board (US). The study was conducted by Merck Sharp & Dohme Corp., a subsidiary of Merck & Co., Inc., Kenilworth, NJ, USA. The sponsor was involved in study design, in the collection, analysis and interpretation of data, in the writing of the report, and in the decision to submit the article for publication.

Participants included men and women 18 years or older with BMI < 40 m^2^/kg and ASA Physical Class 3 or 4 as determined by the investigator (independent of the BMI ≥40 kg/m^2^ criterion) with planned surgical procedures involving moderate or deep neuromuscular block with either rocuronium or vecuronium [22]. Exclusion criteria were: pacemaker or implantable cardioverter-defibrillator precluding assessment of bradycardia or arrhythmias; plan not to reverse neuromuscular block at procedure end; neuromuscular disorder affecting neuromuscular block or assessments; severe renal insufficiency (defined as calculated CrCl < 30 mL/min by Cockroft-Gault); history or family history of malignant hyperthermia; known or suspected allergy to peri-operative medications; toremifene application within 24 h (before or after) study drug administration; pregnant, attempting to become pregnant, or lactating.

**Fig. 1:**
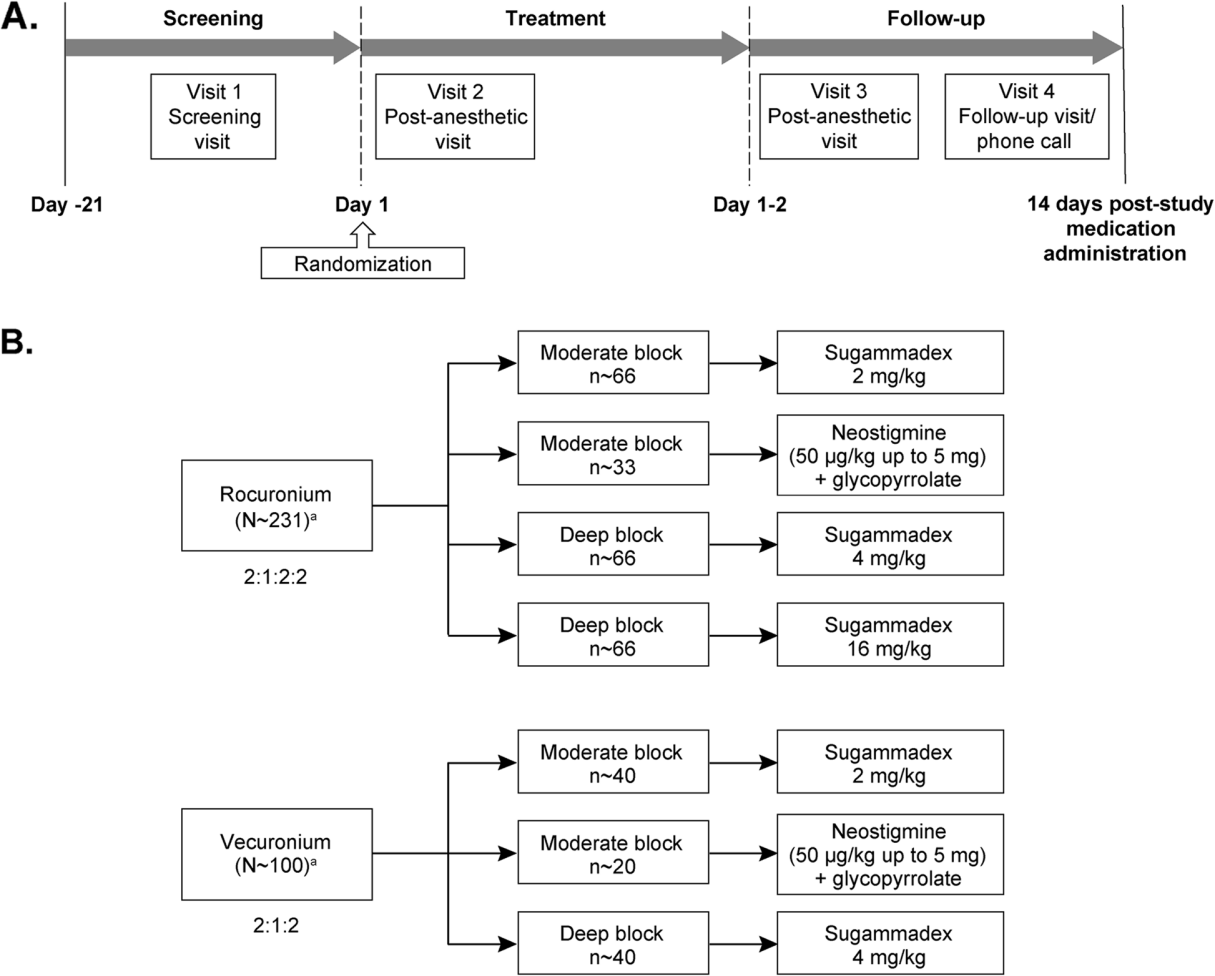
Description of (**A**) study design and (**B**) randomization scheme ^a^ Participants were also stratified by neuromuscular blocking agent, rocuronium or vecuronium

The trial consisted of four visits (Fig. 1): screening visit, peri-anesthetic visit, post-anesthetic visit, and a follow-up safety contact occurring 14 days post study medication. The investigator specified the intended use of rocuronium and vecuronium as appropriate for the type of surgery (provided both strata remained open) at enrollment. The protocol did not specify anesthetic agents for induction or maintenance. Depending on treatment assignment, participants were maintained, according to standard clinical practice, in either moderate neuromuscular block (targeting train-of-four counts between 1 and 3) or deep neuromuscular block (targeting post-tetanic counts < 5) intraoperatively until the time of reversal. Neuromuscular monitoring was performed either qualitatively or quantitatively using any available technique depending on the standard of the respective study center.

**Fig. 2:**
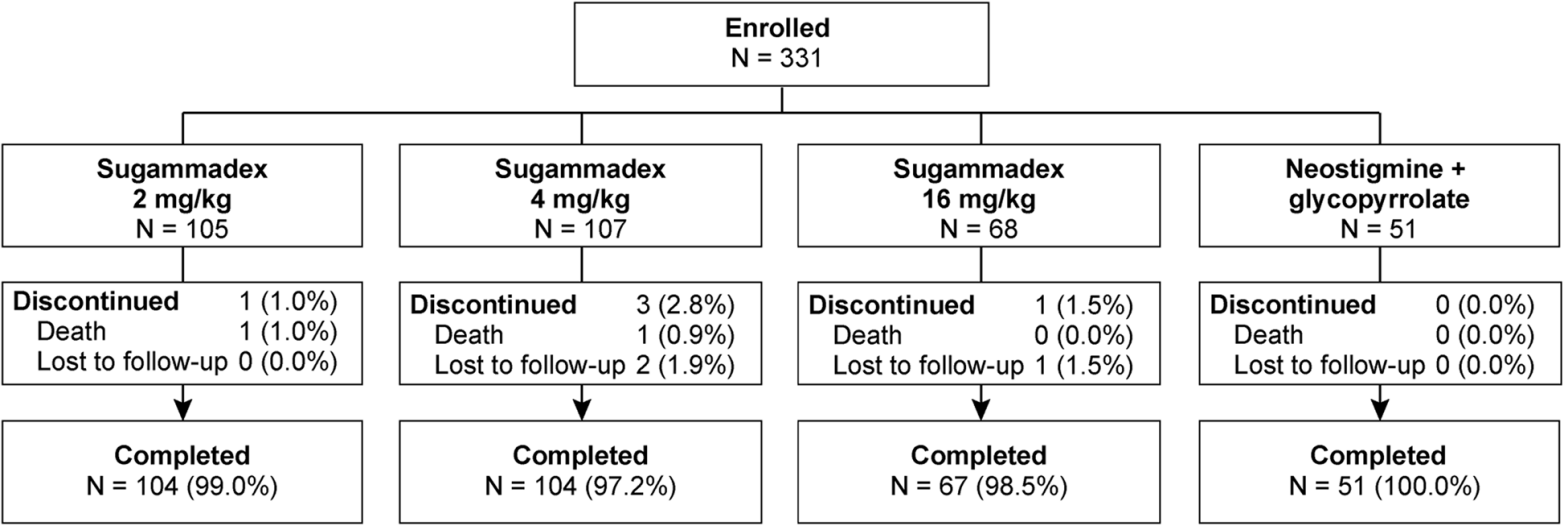
Participant disposition flow chart

An automated Interactive Voice Response System was used for randomization. Treatment assignment determined the depth of neuromuscular block and study medication for its reversal, randomized among seven maintenance/reversal combinations, stratified by choice of rocuronium or vecuronium (Fig. 2). Vecuronium enrollment was capped at 30% and the target number of randomized participants in the ASA Physical Class 4 stratum was approximately 25%. Within the rocuronium stratum, participants were randomized to one of four treatment groups in a 2:1:2:2 ratio as follows (N = ~ 231): 1) Moderate neuromuscular block and reversal with sugammadex 2 mg/kg; 2) Moderate neuromuscular block and reversal with neostigmine (50 μg/kg up to 5 mg maximum dose) plus glycopyrrolate (10 μg/kg up to 1 mg maximum dose) hereafter referred to as neostigmine/glycopyrrolate; 3) Deep neuromuscular block and reversal with sugammadex 4 mg/kg; 4) Deep neuromuscular block and reversal with sugammadex 16 mg/kg. Sugammadex 16 mg/kg, the dose labeled for use for reversal of high-dose rocuronium in an urgent setting, was evaluated in the context of deep block in this study [24]. Within the vecuronium stratum, participants were randomized to one of three treatment groups in a 2:1:2 ratio as follows (N = ~ 100; i.e., ~ 30% of total population): 1) Moderate neuromuscular block and reversal with sugammadex 2 mg/kg; 2) Moderate neuromuscular block and reversal with neostigmine/glycopyrrolate; 3) Deep neuromuscular block and reversal with sugammadex 4 mg/kg. Unlike rocuronium, vecuronium is not indicated for high dose use in rapid sequence induction; therefore, the vecuronium stratum contains no 16 mg/kg sugammadex arm as this dose of sugammadex is only indicated for reversal of high dose rocuronium.

The anesthesiologist was blinded to the reversal agent in the moderate block arms. In the deep block arms, the anesthesiologist was blinded to the dose of sugammadex. The study had a safety assessor, separate from the anesthesiologist, who was blinded to study medication assignment, depth of neuromuscular block, and drug preparation record. Induction and maintenance of anesthesia proceeded per usual practice. After the last dose of neuromuscular block, participants received the reversal agent intravenously via 2 syringes in masked fashion as a bolus within 5 min detection of reappearance of train-of-four count =2 with a lower limit of 1 and upper limit of 4 counts (in moderate block participants) or post-tetanic count of ≥1 and a train-of-four count of 0 (in deep block participants).

### Study endpoints

The primary safety outcomes compared incidences of TE arrhythmias, including sinus bradycardia, sinus tachycardia and other cardiac arrhythmias, for each of the sugammadex groups vs neostigmine/glycopyrrolate. For arrhythmia detection, continuous electrocardiogram monitoring began ≥5 min before study medication administration and lasted ≥30 min after study medication administration. An event was included in the primary analysis if it occurred within 35 min after administration of the study medication. The proportion of participants with each of the following TE arrhythmias, sustained for ≥1 min after administration of study medication, were compared: sinus bradycardia, defined as a heart rate < 60/min or any decrease by more than 20% below baseline; sinus tachycardia, defined as a heart rate ≥ 100/min or any increase by more than 20% above baseline; and other arrhythmias, defined as a new or worsened arrhythmia, e.g., atrial tachycardia or fibrillation. Pre-specified ECIs included selected non-serious and serious AEs occurring from treatment allocation / randomization through 14 days following cessation of treatment as follows: clinically relevant (CR) arrhythmias, hypersensitivity, anaphylaxis, liver transaminase elevations (i.e., aspartate aminotransferase and alanine aminotransferase ≥3-times upper limit of normal; total bilirubin ≥2-times upper limit of normal; alkaline phosphatase < 2-times upper limit of normal) and CR arrhythmias, defined as those necessitating intervention, as determined by the blinded investigator.

For this study, “treatment emergent” (TE) events refers to any of the previously defined deviations in the electrocardiogram from a regular sinus rhythm that emerged in the time period as defined, following administration of study drug (NMBA reversal agent to treat NMB). In this context, use of the word “treatment” in TE terminology refers only to NMB reversal agent study drug, and does not refer to whether treatment of any kind was carried out for TE arrhythmia events. The term TE is therefore independent of a possible treatment for the TE event itself. In this way, all TE events were objectively identified and included in the analyses of endpoints. This approach avoided subjectivity on the part of the treating physician to decide whether an event had occurred based on a clinical decision of whether or not to treat the arrhythmia event.

An external clinical adjudication committee of anesthesia and allergy experts, blinded to treatment, classified potential cases of hypersensitivity and/or anaphylaxis. The general safety profile of sugammadex also was assessed by monitoring of AEs up to 7 days post-treatment and comparing the incidences of specific AEs, by system organ classes and laboratory/vital sign values by predefined limits of changes in one or more of the treatment groups. A supplemental summary of all AEs occurring up to 14 days post administration of study medication also was provided.

### Statistical analysis

Safety analyses were based on the All Participants as Treated Population which included all randomized participants who received at least one dose of study medication. A tiered approach was applied to the safety analyses. The primary safety endpoints (i.e., proportion of participants with TE cardiac arrythmias) were subject to inferential testing for statistical significance with 95% confidence intervals for between-group comparisons. Secondary safety parameters (e.g., CR cardiac arrythmias adjudicated hypersensitivity and anaphylaxis, and some other supportive safety parameters) were assessed via point estimates with 95% confidence intervals provided for between-group comparisons; only point estimates by treatment group were provided for the remainder of the safety parameters. Between-group comparisons were performed for each dose group vs neostigmine/glycopyrrolate pooled across rocuronium and vecuronium stratum. *P*-value significance testing and 95% confidence intervals for between-group comparisons used the stratified Miettinen and Nurminen method with rocuronium and vecuronium and with ASA Physical Class as stratification factors and were provided to guide clinical interpretation of the results [25]. Since no adjustments were made for multiple treatment comparisons, the nominal *P*-values should be interpreted with caution. All statistical tests were conducted at the α = 0.05 (2-sided) level.

## Results

Twenty seven sites in 4 countries screened 393 participants, of whom 331 were enrolled and randomized (Fig. 2). Of those randomized, 326 completed all protocol visits. Participants distributed evenly by demographic characteristics across treatment groups (Table 1). Treated participants were 79 years old (median); 67% were ≥ 65 years; mean BMI was 28.6 kg/m^2^; 40% were female; majority were white non-Hispanic. There was a slight gender imbalance in the neostigmine/glycopyrrolate group that arose by chance following treatment randomization. Cholecystectomy (6%) and prostatectomy (5%) were the most frequently performed procedures. Pre-existing co-morbid conditions displayed adequate balance across groups; overall 73% had hypertension, 27% hyperlipidemia, 25% coronary artery disease, 24% gastroesophageal reflux disease. The most frequently used anesthetics across all treatment groups were propofol, lidocaine and fentanyl administered intravenously, and sevoflurane administered as an inhalant. No clinically meaningful imbalances in the types of anesthetics used was observed across the treatment groups (data not shown), mitigating the concern that differences in types of anesthesia administered may have impacted the outcomes seen in this study. There were three deaths in the study: 2 who died post-operatively and 1 subject in the sugammadex 4 mg/kg group who did not receive study intervention. None of these deaths were deemed related to study medication by the investigators.

**Table 1:** Participant demographics and baseline characteristics. All Participants as Treated

Characteristic	Sugammadex 2 mg/kg *N* = 105	Sugammadex 4 mg/kg *N* = 107	Sugammadex 16 mg/kg *N* = 68	Neostigmine/Glycopyrrolate *N* = 51
**Gender**				
Male	59 (56.2)	68 (63.6)	39 (57.4)	36 (70.6)
Female	46 (43.8)	39 (36.4)	29 (42.6)	15 (29.4)
**Age** (yrs): Median (Range)	70 (35 to 87)	70 (24 to 92)	71 (44 to 90)	68 (35 to 89)
< 65, n (%)	28 (27)	41 (38)	20 (29)	19 (37)
≥65 to ≤74, n (%)	39 (37)	29 (27)	25 (37)	20 (39)
> 74, n (%)	38 (36)	37 (35)	23 (34)	12 (24)
**BMI:** Mean ± SD (kg/m^2^)	28.1 ± 5.5	28.4 ± 5.3	28.6 ± 5.0	29.3 ± 5.4
Median (range)	27.1 (15.8 to 39.6)	28.4 (16.8 to 39.8)	28.5 (16.4 to 39.0)	28.9 (15.7 to 39.0)
< 30 kg/m^2^, n (%)	67 (63.8)	65 (60.7)	41 (60.3)	26 (51.0)
≥30 to < 40 kg/m^2^, n (%)	38 (36.2)	42 (39.3)	27 (39.7)	25 (49.0)
**ASA Class:** ASA Class 3, n (%)	79 (75.2)	79 (73.8)	51 (75.0)	38 (74.5)
ASA Class 4, n (%)	26 (24.8)	28 (26.2)	17 (25.0)	13 (25.5)
**Creatinine Clearance**^†^ (mL/min)				
Mean ± SD	88.5 ± 33.9	94.5 ± 40.6	92.8 ± 49.2	101.5 ± 46.3
Median (range)	82.6 (27.4 to 176.0)	91.1 (29.3 to 227.5)	83.8 (30.1 to 368.0)	91.3 (39.7 to 268.9)
≤30, n (%)	1 (1.0)	1 (0.9)	0	0
> 30 to ≤50, n (%)	6 (5.7)	10 (9.3)	4 (5.9)	2 (3.9)
> 50 to ≤80, n (%)	42 (40.0)	33 (30.8)	25 (36.8)	16 (31.4)
> 80, n (%)	52 (49.5)	54 (50.5)	34 (50.0)	26 (51.0)
Missing, n (%)	4 (3.8)	9 (8.4)	5 (7.4)	7 (13.7)
**Rocuronium**, n (%)	65 (61.9)	66 (61.7)	68 (100)	32 (62.7)
Mean ± SD, (mg/kg)	1.14 ± 0.62	1.60 ± 0.76	1.60 ± 0.96	1.01 ± 0.50
Median (range), (mg/kg)	1.01 (0.36 to 3.23)	1.45 (0.39 to 3.92)	1.33 (0.43 to 5.44)	0.83 (0.33 to 2.14)
**Vecuronium** (mg/kg), n (%)	40 (38.1)	41 (38.3)	0	19 (37.3)
Mean ± SD, (mg/kg)	0.14 ± 0.08	0.26 ± 0.27	n.a.	0.14 ± 0.07
Median (range), (mg/kg)	0.12 (0.05 to 0.49)	0.19 (0.06 to 1.73)	n.a.	0.13 (0.05 to 0.34)

*Abbreviations*: *NMBA* Neuromuscular blocking agent, *SD* Standard deviation, *n.a* Not applied

^†^Creatinine clearance based on Cockcroft-Gault formula

TE cardiac events occurred infrequently across the groups (Table 2). The incidence of TE sinus bradycardia was significantly lower in the sugammadex 2 mg/kg group vs neostigmine/glycopyrrolate (*P* = 0.026). The incidence of TE sinus tachycardia was significantly lower in the sugammadex 2 and 4 mg/kg groups vs neostigmine/glycopyrrolate (*P* = 0.007 and 0.036, respectively). No significant differences in other TE cardiac arrhythmias were seen between sugammadex and neostigmine/glycopyrrolate intervention groups. The between-group differences in the incidences of TE cardiac arrhythmias seen in the overall population were generally consistent when analyzed across ASA Physical Class status (3 and 4: Table 3) and NMBA stratum (rocuronium, vecuronium; Table 4).

**Table 2:** Summary of TE arrhythmias in overall population. All Participants as Treated

Treatment	n (%)	Difference in %^a^ (95% CI)	*P*-value
TE Sinus Bradycardia			
Neostigmine/glycopyrrolate, *n* = 51	4 (7.8)	(ref.)	
Sugammadex 2 mg/kg, *n* = 105	1 (1.0)	− 6.7 (− 17.5, − 0.8)	0.026
Sugammadex 4 mg/kg, *n* = 107	2 (1.9)	− 6.2 (− 17.3, 0.2)	0.058
Sugammadex 16 mg/kg, *n* = 68	5 (7.4)	− 2.0 (− 17.8, 8.6)	0.730
TE Sinus Tachycardia			
Neostigmine/glycopyrrolate, n = 51	11 (21.6)	(ref.)	
Sugammadex 2 mg/kg, n = 105	7 (6.7)	− 14.9 (− 28.8, − 4.0)	0.007
Sugammadex 4 mg/kg, n = 107	10 (9.3)	− 12.2 (− 26.1, − 0.8)	0.036
Sugammadex 16 mg/kg, n = 68	6 (8.8)	− 9.9 (− 27.6, 3.6)	0.158
Other TE Cardiac Arrhythmias			
Neostigmine/glycopyrrolate, n = 51	1 (2.0)	(ref.)	
Sugammadex 2 mg/kg, n = 105	1 (1.0)	−0.9 (− 9.4, 4.0)	0.637
Sugammadex 4 mg/kg, n = 107	0	− 2.1 (− 10.6, 1.5)	0.134
Sugammadex 16 mg/kg, n = 68	1 (1.5)	−1.7 (− 14.7, 5.8)	0.577

^a^Differences are calculated using the stratified Miettinen and Nurminen method with NMBA and ASA Physical Class strata as factors. *ASA* American Society of Anesthesiologists, *CI* Confidence interval, *TE* Treatment-emergent

**Table 3:** Summary of TE arrhythmias analyzed by ASA Physical Class stratum. All Participants as Treated

Treatment	ASA Physical Class 3			ASA Physical Class 4		
	n (%)	Estimate (95% CI)	*P*-value	n (%)	Estimate (95% CI)	*P*-value
TE Sinus Bradycardia						
Neostigmine/glycopyrrolate, *n* = 38^a^, 13^b^	2 (5.3)	(ref.)		2 (15.4)	(ref.)	
Sugammadex 2 mg/kg, n = 79^a^, 26^b^	1 (1.3)	−4.0 (−16.2, 2.5)	0.202	0	−15.4 (− 42.6, − 0.9)	0.043
Sugammadex 4 mg/kg, *n* = 79^a^, 28^b^	1 (1.3)	− 4.0 (− 16.2, 2.5)	0.202	1 (3.6)	−11.8 (−39.6, 5.9)	0.182
Sugammadex 16 mg/kg, n = 51^a^, 17^b^	4 (7.8)	2.6 (− 10.5, 14.3)	0.633	1 (5.9)	−9.5 (− 38.1, 15.2)	0.398
TE Sinus Tachycardia						
Neostigmine/glycopyrrolate, n = 38	7 (18.4)	(ref.)		4 (30.8)	(ref.)	
Sugammadex 2 mg/kg, n = 79	5 (6.3)	−12.1 (−27.8, −0.3)	0.044	2 (7.7)	−23.1 (−51.7, 1.2)	0.063
Sugammadex 4 mg/kg, n = 79	10 (12.7)	−5.8 (− 22.1, 7.4)	0.409	0	−30.8 (− 57.9, − 12.5)	0.002
Sugammadex 16 mg/kg, n = 51	5 (9.8)	−8.6 (− 25.1, 5.9)	0.242	1 (5.9)	−24.9 (− 53.6, 2.8)	0.075
Other TE Cardiac Arrhythmias						
Neostigmine/glycopyrrolate, n = 38	0	(ref.)		1 (7.7)	(ref.)	
Sugammadex 2 mg/kg, n = 79	0	0.0 (−9.3, 4.7)	> 0.999	1 (3.8)	−3.8 (− 30.4, 13.0)	0.612
Sugammadex 4 mg/kg, n = 79	0	0.0 (−9.3, 4.7)	> 0.999	0	−7.7 (−33.7, 5.3)	0.142
Sugammadex 16 mg/kg, n = 51	0	0.0 (−9.3, 7.1)	> 0.999	1 (5.9)	−1.8 (− 29.0, 21.3)	0.846

^a^Number of participants in ASA Physical Class 3 stratum; ^b^number of participants in ASA Physical Class 4 stratum; *ASA* American Society of Anesthesiologists, *CI* Confidence interval, *TE* Treatment-emergent

**Table 4:** Summary of TE arrhythmias analyzed by NMBA stratum. All Participants as Treated

Treatment	Rocuronium			Vecuronium		
	n (%)	Estimate (95% CI)	*P*-value	n (%)	Estimate (95% CI)	*P*-value
TE Sinus Bradycardia						
Neostigmine/glycopyrrolate, *n* = 32^a^, 19^b^	3 (9.4)	(ref.)		2 (15.4)	(ref.)	
Sugammadex 2 mg/kg, *n* = 65^a^, 40^b^	0	−9.4 (−24.3, −3.2)	0.013	0	−2.8 (− 22.6, 8.7)	0.587
Sugammadex 4 mg/kg, *n* = 66^a^, 41^b^	2 (3.0)	−6.3 (− 21.6, 3.0)	0.183	1 (3.6)	−5.3 (− 24.9, 3.8)	0.142
Sugammadex 16 mg/kg, *n* = 68^a^	5 (7.4)	− 2.0 (− 17.7, 8.9)	0.729	N/A	N/A	N/A
TE Sinus Tachycardia						
Neostigmine/glycopyrrolate, n = 32^a^, 19^b^	6 (18.8)	(ref.)		4 (30.8)	(ref.)	
Sugammadex 2 mg/kg, n = 65^a^, 40^b^	6 (9.2)	−9.5 (−27.2, 4.2)	0.183	2 (7.7)	−23.8 (−46.9, −7.1)	0.005
Sugammadex 4 mg/kg, n = 66^a^, 41^b^	7 (10.6)	−8.1 (− 25.9, 5.8)	0.267	0	−19.0 (− 42.7, −0.3)	0.046
Sugammadex 16 mg/kg, n = 68^a^	6 (8.8)	− 9.9 (− 27.5, 3.5)	0.156	N/A	N/A	N/A
Other TE Cardiac Arrhythmias						
Neostigmine/glycopyrrolate, n = 32^a^, 19^b^	1 (3.1)	(ref.)		1 (7.7)	(ref.)	
Sugammadex 2 mg/kg, n = 65^a^, 40^b^	0	−3.1(−15.8, 2.6)	0.154	1 (3.8)	2.5 (−14.7, 13.0)	0.491
Sugammadex 4 mg/kg, n = 66^a^, 41^b^	0	−3.1 (− 15.8, 2.5)	0.151	0	0.0 (−17.1, 8.7)	> 0.999
Sugammadex 16 mg/kg, n = 68^a^	1 (1.5)	−1.7 (− 14.5, 5.3)	0.583	N/A	N/A	N/A

^a^Number of participants in rocuronium stratum; ^b^Number of participants in vecuronium stratum; *CI* Confidence interval, *N/A* Not applicable, *NMBA* Neuromuscular blocking agent, *TE* Treatment-emergent

Overall, the numbers/percentages of participants with ECIs were low in all the intervention groups up to 7 days post-treatment (Table 5). No clinically meaningful differences were observed between groups with respect to the ECIs of CR bradycardia, CR tachycardia and other CR cardiac arrhythmias. No cases of adjudicated anaphylaxis or hypersensitivity reactions and no drug-induced liver injury were reported at any time during this study. The incidences of elevated ALT, AST, bilirubin and alkaline phosphates were low and similar across the intervention groups. No participants met the creatinine clearance predetermined criterion of < 30 mL/min up to 14 days post-treatment. No clinically meaningful findings relating to the administration of sugammadex were observed in the mean changes of vital sign assessments or incidences of vital sign findings that met predetermined criteria.

**Table 5:** Selected AEs of clinical interest up to 7 days post-treatment. All Participants as Treated

	n (%)	Difference in %^a^ (95% CI)
With ≥ ECIs^b^		
Neostigmine/glycopyrrolate, *n* = 51	2 (3.9)	(ref.)
Sugammadex 2 mg/kg, *n* = 105	2 (1.9)	−2.1 (− 11.8, 3.8)
Sugammadex 4 mg/kg, *n* = 107	6 (5.6)	1.8 (−8.1, 8.4)
Sugammadex 16 mg/kg, *n* = 68	5 (7.4)	1.1 (−13.5, 11.1)
Clinically Relevant Bradycardia		
Neostigmine/glycopyrrolate, *n* = 51	1 (2.0)	(ref.)
Sugammadex 2 mg/kg, n = 105	0	−2.0 (−10.5, 1.6)
Sugammadex 4 mg/kg, n = 107	3 (2.8)	0.9 (− 7.8, 6.6)
Sugammadex 16 mg/kg, n = 68	0	− 3.1 (− 15.9, 2.4)
Clinically Relevant Tachycardia		
Neostigmine/glycopyrrolate, *n* = 51	0	(ref.)
Sugammadex 2 mg/kg, *n* = 105	2 (1.9)	1.9 (−5.3, 6.8)
Sugammadex 4 mg/kg, *n* = 107	2 (1.9)	1.8 (− 5.4, 6.6)
Sugammadex 16 mg/kg, n = 68	4 (5.9)	5.9 (− 5.0, 14.3)
Other Clinically Relevant Cardiac Arrhythmia		
Neostigmine/glycopyrrolate, n = 51	1 (2.0)	(ref.)
Sugammadex 2 mg/kg, n = 105	0	−2.0 (−10.5, 1.6)
Sugammadex 4 mg/kg, n = 107	1 (0.9)	−1.0 (− 9.5, 3.5)
Sugammadex 16 mg/kg, *n* = 68	1 (1.5)	−1.7 (− 14.6, 5.4)

^a^Differences are calculated using the stratified Miettinen and Nurminen method with NMBA and ASA Physical Class strata as factors. ^b^No AEs of adjudicated hypersensitivity, adjudicated anaphylaxis, or drug-induced liver injury were reported up to 7 days post-treatment; *ASA* American Society of Anesthesiologists, *AE* Adverse event, *CI* Confidence interval, *ECI* Events of clinical interest

The numbers/percentages of participants with AEs and drug-related AEs reported up to 7 days post-treatment were similar across the 4 intervention groups (Table 6). Although there were numerical differences in the incidence of serious adverse events (SAEs) between the sugammadex and neostigmine/glycopyrrolate intervention groups, there were no imbalances (based on the 95% CIs) and the differences were not clinically meaningful. One drug-related SAE in the sugammadex 16 mg/kg group was reported (i.e., cardiac arrest Day 1), resolved, and no treated participants discontinued due to an AE. One subject in each of the sugammadex 2- and 4-mg/kg groups had a SAE that resulted in death. The subject in the sugammadex 4 mg/kg group had a SAE of cardiac failure on Day 2 and the subject in the sugammadex 2 mg/kg group had a SAE of cardiac arrest on Day 9. No clinically meaningful differences were observed between the sugammadex and neostigmine/glycopyrrolate intervention groups in the analysis of specific AEs (incidence of ≥4). Consistent with expectations in participants undergoing surgery, AEs of procedural pain (45.6 to 54.3%) and incision site pain (17.6 to 29.0%) were reported most frequently across all groups.

**Table 6:** Overall AE summary up to 7 days post-treatment. All Participants as Treated

	n (%)	Difference in %^a^ (95% CI)
Patients with ≥1 AEs		
Neostigmine/glycopyrrolate, n = 51	45 (88.2)	(ref.)
Sugammadex 2 mg/kg, n = 105	99 (94.3)	6.2 (−2.6, 18.5)
Sugammadex 4 mg/kg, *n* = 107	95 (88.8)	0.5 (− 9.3, 13.1)
Sugammadex 16 mg/kg, *n* = 68	63 (92.6)	2.0 (− 8.4, 17.7)
Patients with ≥1 drug-related^b^ AEs		
Neostigmine/glycopyrrolate, n = 51	4 (7.8)	(ref.)
Sugammadex 2 mg/kg, n = 105	4 (3.8)	−4.1 (− 15.3, 3.3)
Sugammadex 4 mg/kg, n = 107	5 (4.7)	− 3.1 (− 14.3, 4.3)
Sugammadex 16 mg/kg, n = 68	2 (2.9)	−6.4 (−21.7, 2.6)
Patients with ≥1 serious AEs^c^		
Neostigmine/glycopyrrolate, n = 51	3 (5.9)	(ref.)
Sugammadex 2 mg/kg, n = 105	12 (11.4)	5.6 (−5.5, 14.3)
Sugammadex 4 mg/kg, n = 107	8 (7.5)	1.5 (−9.2, 9.3)
Sugammadex 16 mg/kg, n = 68	7 (10.3)	0.9 (−15.1, 12.7)
Patients with ≥1 serious drug-related^b^ AEs		
Neostigmine/glycopyrrolate, n = 51	0 (0.0)	(ref.)
Sugammadex 2 mg/kg, n = 105	0	0.0 (−7.2, 3.6)
Sugammadex 4 mg/kg, n = 107	0	0.0 (− 7.2, 3.6)
Sugammadex 16 mg/kg, n = 68	1 (1.5)	1.5 (−9.5, 8.0)

^a^Differences are calculated using the stratified Miettinen and Nurminen method with NMBA and ASA Physical Class strata as factors. ^b^Rated as possibly, probably or definitely related to study medication by the study investigator

^c^Including 1 death in the 2 mg/kg and 4 mg/kg sugammadex groups each. *AE* Adverse event, *ASA* American Anesthesiology Association, *CI* Confidence interval, *NMBA* Neuromuscular binding agent

## Discussion

Results of this dedicated randomized study in ASA Physical Class 3 or 4 participants demonstrate that treatment with sugammadex compared with neostigmine/glycopyrrolate did not lead to clinically meaningful differences in heart rate or rhythm changes. Overall, TE cardiac events were generally low and less frequent than the incidences observed with the comparator neostigmine/glycopyrrolate. Compared with neostigmine/glycopyrrolate, the incidence of TE sinus bradycardia was significantly lower with sugammadex 2 mg/kg and the incidence of TE sinus tachycardia was significantly lower with sugammadex 2 mg/kg and 4 mg/kg. No significant differences in other cardiac arrythmias (TE or CR) were seen between sugammadex groups and neostigmine/glycopyrrolate.

The overall incidences and type of AEs were generally similar across the intervention groups, including drug-related AEs and SAEs (reported up to 7 days post-treatment). Two deaths occurred among participants who received study medication in this study, 1 each in the sugammadex 4 mg/kg group and 2 mg/kg groups (reporting SAEs of cardiac arrest and cardiac failure, respectively); both SAEs were assessed by the investigator as not related to study medication. The incidences of ECIs were low overall in this study across all interventions. Treatment with sugammadex did not result in any reports of hypersensitivity, anaphylaxis, or liver toxicity (at any timepoint). Further, the overall safety profile was similar when comparing participants by ASA Physical Class (3 vs 4) and NMBA (rocuronium vs vecuronium).

Taken together, these findings demonstrate that the safety profile of sugammadex in ASA Physical Class 3 or 4 patients does not meaningfully differ from the known profile established in the predominantly studied ASA Physical Class 1 or 2 populations [6–10]. Theoretical concerns that ASA Physical Class 3 and 4 participants may be at increased risk for labeled risks associated with sugammadex administration, namely risks for bradycardia or hypersensitivity/anaphylaxis, did not materialize in the current study, supporting the use of sugammadex for reversal of rocuronium- or vecuronium-induced neuromuscular block in this important, medically vulnerable population.

While this study was specifically designed to detect treatment-related differences in the incidences of treatment-emergent (TE) sinus bradycardia, TE sinus tachycardia, and other TE cardiac arrhythmias, a possible limitation of this study is the relative lack of powering for characterization of CR arrhythmias incidences. In this study, all TE arrhythmia events detected were evaluated by the investigator for potential clinical relevance and few TE events were deemed CR, an outcome which may limit further interpretation of the results with regard to risk for CR events. However, consistent with the results of this trial in higher risk ASA Class 3 or 4 participants, CR events associated with sugammadex administration have not been commonly observed in randomized clinical trials [16–19], but rather in infrequent pharmacovigilance reports [2, 21], suggesting it would be infeasible to design a prospective study specifically for that purpose.

A second potential limitation of the study is the lower sample size allocated to evaluation of the sugammadex 16 mg/kg and neostigmine/glycopyrrolate groups in the overall study population (*n* = 68, *n* = 51, respectively), lowering the precision for characterization of CR events in these groups. Of note, however, sugammadex 16 mg/kg is only intended for use in an emergency setting for urgent reversal of rocuronium [17], where it can be life-saving, a benefit arguably outweighing a potential risk of CR arrhythmia.

Another potential limitation of this study involves the use of the ASA physical status grading system, which is known to have low inter-rater reliability based on the experience level of the anesthesiologist assigning the classification [26]. On average, more experienced anesthesiologists are less accurate in classifying patients compared to less experienced colleagues. While the current study did not control for the potential bias of low inter-rater reliability of the ASA grading system, principal investigators and/or appropriately trained personnel assigned to the study were responsible for evaluating and classifying each patient prior to surgery. Further, the prespecified primary analysis pooled findings across ASA Class 3 and 4 strata thus enabling a general assessment of the relative safety profile of sugammadex vs neostigmine/glycopyrrolate in a broad range of at-risk patients. Nevertheless, caution should be used when drawing conclusions about the relative cardiac safety of sugammadex vs neostigmine/glycopyrrolate between Grade 3 vs Grade 4 patients.

## Conclusion

In conclusion, the results of this study support the overall favorable safety profile, inclusive of cardiac safety parameters, of sugammadex for reversal of rocuronium- or vecuronium-induced moderate and deep neuromuscular block in ASA Class 3 or 4 participants.

## Supplementary Information

**Additional file 1.** Protocol 145.

**Additional file 2.** CONSORT checklist.

## Data Availability

Merck Sharp & Dohme Corp., a subsidiary of Merck & Co., Inc., Kenilworth, NJ, USA’s data sharing policy, including restrictions, is available at http://engagezone.msd.com/ds_documentation.php. Requests for access to the clinical study data can be submitted through the EngageZone site or via email to dataaccess@merck.com.

## Abbreviations

AE: Adverse event
ASA: American Society of Anesthesiologists
BMI: Body mass index
CONSORT: Consolidated Standards of Reporting Trials
CR: Clinically relevant
ECI: Events of clinical interest
NMBA: Neuromuscular blockade agent
SAE: Serious adverse event
SD: Standard deviation
TE: Treatment-emergent
