# Supplementary material for: A randomized trial evaluating the safety profile of sugammadex in high surgical risk ASA physical class 3 or 4 participants
Source: BMC Anesthesiol. 2021 Oct 28;21:259. doi: 10.1186/s12871-021-01477-5 (PMC8555093; doi:10.1186/s12871-021-01477-5)
Supplement: Supplementary file 1 — Additional file 1. Protocol 145. [file 12871_2021_1477_MOESM1_ESM.pdf]

Supplementary Information for

Title: **A randomized trial evaluating the safety profile of sugammadex in high surgical risk ASA Physical Class 3 or 4 participants**

W. Joseph Herring, Yuki Mukai, Aobo Wang, Jeannine Lutkiewicz, John F. Lombard, Li Lin, Molly Watkins, David M. Broussard, Manfred Blobner

Table of Contents

Additional file 1..2

Additional file 2..84

Merck & Co., Inc. policy on posting of redacted study protocols on journal websites is described in Guidelines for Publication of Clinical Trials in the Scientific Literature on the www.merck.com website.

For publicly posted protocols, the Company redacts content that contains proprietary and private information.

This report may include approved and non-approved uses, formulations, or treatment regimens. The results reported may not reflect the overall profile of a product. Before prescribing any product mentioned in this report, healthcare professionals should consult local prescribing information for the product approved in their country.

Copyright © 2021 Merck Sharp & Dohme Corp., a subsidiary of Merck & Co., Inc.
All Rights Reserved. Not for regulatory or commercial use.

THIS PROTOCOL AMENDMENT AND ALL OF THE INFORMATION RELATING TO IT ARE CONFIDENTIAL AND PROPRIETARY PROPERTY OF MERCK SHARP & DOHME CORP., A SUBSIDIARY OF MERCK & CO., INC., WHITEHOUSE STATION, NJ, U.S.A.

SPONSOR:

Merck Sharp & Dohme Corp., a subsidiary of Merck & Co., Inc.
(hereafter referred to as the Sponsor or Merck)

One Merck Drive
P.O. Box 100
Whitehouse Station, New Jersey, 08889-0100, U.S.A.

Protocol-specific Sponsor Contact information can be found in the Investigator Trial File Binder (or equivalent).

TITLE:

A Phase 4 Randomized, Active-Comparator Controlled Clinical Trial to Study the Safety of Sugammadex (MK-8616) for the Reversal of Neuromuscular Blockade Induced by Either Rocuronium Bromide or Vecuronium Bromide in American Society of Anesthesiologists (ASA) Class 3 or 4 Subjects

IND NUMBER: 68,029
EudraCT NUMBER: 2017-000187-15

TABLE OF CONTENTS

SUMMARY OF CHANGES 9

1.0 TRIAL SUMMARY 15

2.0 TRIAL DESIGN 16

2.1 Trial Design 16

2.2 Trial Diagram 19

3.0 OBJECTIVE(S) & HYPOTHESIS(ES) 20

3.1 Primary Objective(s) & Hypothesis(es) 20

3.2 Secondary Objective(s) & Hypothesis(es) 20

4.0 BACKGROUND & RATIONALE 20

4.1 Background 20

4.1.1 Pharmaceutical and Therapeutic Background 21

4.2 Rationale 21

4.2.1 Rationale for the Trial and Selected Subject Population 21

4.2.2 Rationale for Dose Selection/Regimen 22

4.2.2.1 Rationale for the Use of Comparator 23

4.2.3 Rationale for Endpoints 23

4.2.3.1 Efficacy Endpoints 23

4.2.3.2 Safety Endpoints 23

4.2.3.3 Future Biomedical Research 24

4.3 Benefit/Risk 25

5.0 METHODOLOGY 25

5.1 Entry Criteria 25

5.1.1 Diagnosis/Condition for Entry into the Trial 25

5.1.2 Subject Inclusion Criteria 25

5.1.3 Subject Exclusion Criteria 26

5.2 Trial Treatment(s) 28

5.2.1 Dose Selection 28

5.2.1.1 Dose Selection (Preparation) 28

5.2.2 Timing of Dose Administration 29

5.2.3 Trial Blinding 29

5.3 Randomization 30
5.4 Stratification 31
5.5 Concomitant Medications/Vaccinations (Allowed & Prohibited) 31
5.6 Rescue Medications & Supportive Care 31
5.7 Diet/Activity/Other Considerations 31
5.8 Subject Withdrawal from the Trial 31
5.9 Subject Replacement Strategy 32
5.10 Beginning and End of the Trial 32
5.11 Clinical Criteria for Early Trial Termination 32
6.0 TRIAL FLOW CHART 33
7.0 TRIAL PROCEDURES 36
7.1 Trial Procedures 36
7.1.1 Administrative Procedures 36
7.1.1.1 Informed Consent 36
7.1.1.1.1 General Informed Consent 36
7.1.1.1.2 Consent and Collection of Specimens for Future Biomedical Research 37
7.1.1.2 Inclusion/Exclusion Criteria 37
7.1.1.3 Subject Identification Card 37
7.1.1.4 Medical History 37
7.1.1.5 Prior and Concomitant Medications Review 37
7.1.1.5.1 Prior Medications 37
7.1.1.5.2 Concomitant Medications 37
7.1.1.6 Assignment of Screening Number 38
7.1.1.7 Assignment of Treatment/Randomization Number 38
7.1.1.8 Trial Compliance (Medication) 38
7.1.2 Clinical Procedures/Assessments 38
7.1.2.1 Physical Examination 38
7.1.2.2 Body Weight and Height 39
7.1.2.3 Body Mass Index 39
7.1.2.4 Electrocardiogram 39
7.1.2.5 Vital Signs 39

7.1.2.6 Administration of Neuromuscular Blocking Agents 41
7.1.2.7 Neuromuscular Monitoring.. 41
7.1.3 Laboratory Procedures/Assessments 41
7.1.3.1 Laboratory Safety Evaluations (Hematology and Chemistry) 42
7.1.3.2 Pregnancy Tests 42
7.1.3.3 Future Biomedical Research Samples 43
7.1.4 Other Procedures.. 43
7.1.4.1 Withdrawal/Discontinuation 43
7.1.4.1.1 Withdrawal From Future Biomedical Research 43
7.1.4.1.2 Lost to Follow-up.. 43
7.1.4.1.3 Subject Blinding/Unblinding 44
7.1.4.2 Calibration of Equipment.. 44
7.1.5 Visit Requirements... 44
7.1.5.1 Screening Visit (V1) 45
7.1.5.2 Peri-anesthetic Visit (V2) 45
7.1.5.3 Post-Anesthetic Visit (V3) 47
7.1.5.4 Follow-up Safety Contact (V4).. 48
7.2 Assessing and Recording Adverse Events and Patient/Device Events 48
7.2.1 Definition of an Overdose for This Protocol and Reporting of Overdose to the Sponsor.. 49
7.2.2 Reporting of Pregnancy and Lactation to the Sponsor 50
7.2.3 Immediate Reporting of Adverse Events and Incidents to the Sponsor 50
7.2.3.1 Serious Adverse Events and Incidents 50
7.2.3.2 Events of Clinical Interest.. 52
7.2.4 Evaluating Adverse Events 53
7.2.5 Sponsor Responsibility for Reporting Adverse Events 56
7.3 TRIAL GOVERNANCE AND OVERSIGHT 56
7.3.1 Clinical Adjudication Committee 56
8.0 STATISTICAL ANALYSIS PLAN 56
8.1 Statistical Analysis Plan Summary 57
8.2 Interim Analyses 58
8.3 Responsibility for Analyses/In-House Blinding 58

8.4 Hypotheses/Estimation 58
8.5 Analysis Endpoints 58
8.5.1 Tier 1 Safety Endpoints 58
8.5.2 Tier 2 Safety Endpoints 59
8.6 Analysis Populations 59
8.7 Statistical Methods 59
8.7.1 Statistical Methods for Safety Analyses 60
8.7.2 Demographic and Baseline Characteristics 61
8.8 Multiplicity 61
8.9 Sample Size and Power Calculations 62
8.10 Subgroup Analyses and Effect of Baseline Factors 64
8.11 Compliance (Medication Adherence) 64
8.12 Extent of Exposure 64
9.0 LABELING, PACKAGING, STORAGE AND RETURN OF CLINICAL SUPPLIES 64
9.1 Investigational Product 64
9.2 Packaging and Labeling Information 65
9.3 Clinical Supplies Disclosure 65
9.4 Storage and Handling Requirements 65
9.5 Discard/Destruction/Returns and Reconciliation 66
9.6 Standard Policies 66
10.0 ADMINISTRATIVE AND REGULATORY DETAILS 66
10.1 Confidentiality 66
10.1.1 Confidentiality of Data 66
10.1.2 Confidentiality of Subject Records 66
10.1.3 Confidentiality of Investigator Information 67
10.1.4 Confidentiality of IRB/IEC Information 67
10.2 Compliance with Financial Disclosure Requirements 67
10.3 Compliance with Law, Audit and Debarment 68
10.4 Compliance with Trial Registration and Results Posting Requirements 69
10.5 Quality Management System 70
10.6 Data Management 70

10.7 Publications 70

11.0 LIST OF REFERENCES 71

12.0 APPENDICES 73

12.1 Merck Code of Conduct for Clinical Trials 73

12.2 Collection and Management of Specimens for Future Biomedical Research 75

12.3 Approximate Blood/Tissue Volumes Drawn/Collected by Trial Visit and by Sample Types 79

12.4 List of Abbreviations and Definitions of Terms 80

13.0 SIGNATURES 81

13.1 Sponsor's Representative 81

13.2 Investigator 81

LIST OF TABLES

Table 1 Trial Treatments ... 28
Table 2 Vital Sign Measurements ... 40
Table 3 Laboratory Tests ... 42
Table 4 Evaluating Adverse Events ... 54
Table 5 Analysis Strategy for Safety Parameters ... 61
Table 6 Estimate of Incidence of Any Treatment Emergent Arrhythmia and 95% Upper Confidence Bound Based on Hypothetical Number of Subjects with Any Treatment Emergent Arrhythmia Among 40 and 66 Subjects in the Sugammadex Group ... 63
Table 7 Differences in Incidence in Any Treatment Emergent Arrhythmia between the Sugammadex and Neostigmine Groups (Assuming Two-Sided 5% alpha level with 66 Subjects in Sugammadex Group and 33 Subjects in Neostigmine Group) ... 63
Table 8 Product Descriptions ... 65

LIST OF FIGURES

Figure 1 Trial Design 20

SUMMARY OF CHANGES

PRIMARY REASON FOR THIS AMENDMENT:

Section Number	Section Title	Description of Change	Rationale
6.0	Trial Flow Chart	Update to Trial Flow Chart to allow Visit 1 and Visit 2 to occur on the same day.	To provide flexibility to trial sites.

ADDITIONAL CHANGE(S) FOR THIS AMENDMENT:

Section Number (s)	Section Title (s)	Description of Change (s)	Rationale
2.1	Trial Design	Added:<br>"The screening and peri-anesthetic visits may occur on the same day (see Section 7.1.5.2)."	Text added for consistency with updates to the Trial Flow Chart and Section 7.1.5.2.
2.1<br>4.2.3.2	Trial Design<br>Safety Endpoints	Added additional formatting and minor textual clarifications for safety endpoints: treatment emergent sinus bradycardia, treatment emergent sinus tachycardia, and other treatment emergent cardiac arrhythmias<br>Added: Definition of baseline heart rate	Additional text, formatting and definition of baseline heart rate added for clarity and alignment with Section 8.5.1 Tier 1 Safety Endpoints.<br>Note: the definition of these safety endpoints has <u>**not**</u> changed.
2.2	Trial Diagram	Dose units for neostigmine corrected from 50 μ/kg to 50 μ**g**/kg	Correction to dose units in Figure
2.2<br>6.0	Trial Diagram<br>Trial Flow Chart	Screening Visit (V1) window extended to 21 days	Screening Visit (V1) window extended from 14 to 21 days to allow greater flexibility for trial sites.

Section Number (s)	Section Title (s)	Description of Change (s)	Rationale
2.2<br>6.0	Trial Diagram<br>Trial Flow Chart	Visit 4: Follow-Up Visit/Phone Call occurs 14 days post study medication administration	Clarification provided for the timing of follow up visit/call.
5.1.3	Subject Exclusion Criteria	Added note to exclusion criteria #4:<br>If dialysis or severe renal insufficiency is not known or confirmed in the subject's medical history, a creatinine clearance value should be calculated based upon the serum creatinine values obtained from local labs within the past 2 weeks prior to Visit 1 or local labs performed between V1 and V2 (e.g., pre-operative testing).	Additional clarification provided to assist trial sites with determining subject eligibility.
5.2.2	Timing of Dose Administration	Previous wording stated: Sugammadex or neostigmine administration should not exceed 20 seconds.<br>Updated for clarity: The total duration of administration for all study medication syringes (Dose 1 and Dose 2 syringe(s) as outlined in Study Drug Manual) should not exceed a total of 20 seconds.	Updated this language to clarify that all syringes, not just the syringes containing sugammadex or neostigmine, should be administered within 20 seconds. The standardization of timing for study medication administration across treatment arms is necessary to ensure consistency of dosing across sites and to maintain the treatment blind.

Section Number (s)	Section Title (s)	Description of Change (s)	Rationale
5.2.3	Trial Blinding	The 4th bulleted item regarding BSA responsibilities:<br>• Be required to sign a statement for each subject **(after V4 or discontinuation)** confirming that the blind was maintained (treatment group, **depth of block, drug preparation records**).	Textual update to provide consistency with the BSA statement and other sections of the protocol and to clarify the BSA signs this statement after Visit 4 or subject discontinuation.
6.0<br><br>7.1.3.2<br><br>Appendix 12.3	Trial Flow Chart<br><br>Pregnancy Tests<br><br>Approximate Blood/Tissue Volumes Drawn/Collected by Trial Visit and by Sample Types	Protocol sections updated to show that a serum β-human chorionic gonadotropin (β-hCG) pregnancy test (results analyzed at a local lab) is acceptable for Visit 1 or within 24 hours prior to surgery in place of a urine pregnancy test.	The serum β-hCG pregnancy test was added as an acceptable method of testing for Visit 1 or within 24 hours prior to surgery for sites that use this method of testing as a part of their standard of care process.
7.1.2.2	Body Weight and Height	The measurement of height and weight should be rounded to the nearest whole number.	Clarification

**Section Number (s)**	**Section Title (s)**	**Description of Change (s)**	**Rationale**
7.1.2.4	Electrocardiogram	Added: Historical ECGs done prior to informed consent are not acceptable for the Visit 1 ECG.<br><br>To assist sites with scheduling study procedures, the screening ECG may be performed at Visit 2 if necessary. However, it must be performed prior to induction of anesthesia or prior to administration of study medication.<br><br>Printing of the ECG trace is not required per protocol.	Clarification provided for Visit 1 and Visit 2 ECG requirements.
7.1.2.5	Vital Signs	Added : Table 2 Vital Sign Measurements	Timing and methods for vital sign measurements displayed in table format for additional clarity.
7.1.3.1	Laboratory Safety Evaluations	Changed: "Safety labs should be obtained pre-administration of study medication" to "Safety labs should be obtained pre-administration of **NMBA**"	Correction
7.1.4.1.3	Subject Blinding/ Unblinding	Update:<br><br>Treatment/Vaccine identification information is to be unmasked ONLY if necessary for the welfare of the subject. Every effort should be made not to unblind the subject ~~**unless necessary.**~~	Removal of redundant text

Section Number (s)	Section Title (s)	Description of Change (s)	Rationale
7.1.4.2	Calibration of Equipment	Removed reference to list of critical equipment.	Textual revisions were applied to clarify investigator responsibility for calibration and maintenance of study equipment.
2.1;<br>7.1.5.2	Trial Design<br>Peri-anesthetic Visit (V2)	Updated PTC value from PTC >1, to PTC ≥ 1	Typographical error that has been corrected in order to be consistent with other sections of the protocol.
7.1.5.2	Peri-anesthetic Visit (V2)	Added text to describe the conduct of protocol procedures when Visit 1 and Visit 2 occur on the same day.<br>Section formatting updated (bullets and numbering).	Additional guidance provided on the order of trial procedures in relation to randomization in IRT, and new guidance for when Visit 1 and Visit 2 are performed on the same day.
7.2	Adverse Events	Removed:<br>"All device or patient events that occur after allocation/randomization must be reported by the investigator if they cause the subject to be excluded from the trial, or are the result of a protocol-specified intervention, including but not limited to washout or discontinuation of usual therapy, diet, placebo treatment or a procedure."	Text was not applicable to the trial; the device used for neuromuscular monitoring (peripheral nerve stimulator or TOF-Watch SX®) is only used after subject randomization. Device incidents can only be reported for randomized subjects in this trial.

**Section Number (s)**	**Section Title (s)**	**Description of Change (s)**	**Rationale**
7.2.3.1	Serious Adverse Events and Incidents	Removed:<br>"For the time period beginning at treatment allocation/randomization, any incident, including follow up to an incident, including death due to any cause, that occurs to any subject must be reported within 24 hours to the Sponsor if it causes the subject to be excluded from the trial, or is the result of a protocol-specified intervention, including but not limited to washout or discontinuation of usual therapy, diet, placebo treatment or a procedure.	Text was not applicable to the trial, the device used for neuromuscular monitoring (peripheral nerve stimulator or TOF-Watch SX®) is only used after subject randomization. Device incidents can only be reported for randomized subjects in this trial.
7.3.1	Clinical Adjudication Committee (CAC)	Previous wording stated: Upon identification of an eligible event, the Sponsor will query the site to confirm that the event is eligible for adjudication based on the AE term and the timing of the event.<br>Revised to: Upon identification of such an event, the Sponsor may query the site for additional information.	Removed language to be consistent with process; CAC will determine if an event is eligible for adjudication, not the site.
Throughout protocol	Throughout protocol	Minor typographical updates	For clarity

1.0 TRIAL SUMMARY

Abbreviated Title	Safety of Sugammadex for the Reversal of Neuromuscular Blockade in American Society of Anesthesiologists (ASA) Class 3 or 4 Subjects
Sponsor Product Identifiers	MK-8616<br>Sugammadex
Trial Phase	Phase 4
Clinical Indication	Reversal of neuromuscular blockade
Trial Type	Interventional
Type of control	Active control without placebo
Route of administration	Intravenous
Trial Blinding	Double-blind
Treatment Groups	Within the rocuronium stratum, the treatment groups are:<br>1) Moderate block and reversal with sugammadex 2 mg/kg; or<br>2) Moderate block and reversal with neostigmine (50 µg/kg up to 5 mg maximum dose) + glycopyrrolate (10 µg/kg up to 1 mg maximum dose); or<br>3) Deep block and reversal with sugammadex 4 mg/kg; or<br>4) Deep block and reversal with sugammadex 16 mg/kg<br>Within the vecuronium stratum, the treatment groups are:<br>1) Moderate block and reversal with sugammadex 2 mg/kg; or<br>2) Moderate block and reversal with neostigmine (50 µ g/kg up to 5 mg maximum dose) + glycopyrrolate (10 µg/kg up to 1 mg maximum dose); or<br>3) Deep block and reversal with sugammadex 4 mg/kg;
Number of trial subjects	Approximately 331 subjects will be enrolled.
Estimated duration of trial	The Sponsor estimates that the trial will require approximately 3 years from the time the first subject signs the informed consent until the last subject's last study-related phone call or visit.
Duration of Participation	Each subject will participate in the trial for approximately 30 days from the time the subject signs the Informed Consent Form (ICF) through the final contact. After the screening period, each subject will receive the assigned treatment on their operative day. After treatment, each subject will undergo a post-anesthetic visit a minimum of 4 hours or up to a maximum of 36 hours after administration of study medication. A follow-up safety contact will take place at approximately 14 days post treatment to assess for AEs.
Randomization Ratio	2:1:2:2 for rocuronium and 2:1:2 for vecuronium

A list of abbreviations used in this document can be found in Section 12.4.

2.0 TRIAL DESIGN

2.1 Trial Design

This is a randomized, active comparator-controlled, parallel-group, multi-site, double-blind safety trial of sugammadex for the reversal of neuromuscular blockade (NMB) induced by rocuronium or vecuronium in adult American Society of Anesthesiologists (ASA) Physical Status Class 3 and 4 subjects. The purpose of this study is to characterize the incidence of treatment emergent sinus bradycardia, treatment emergent sinus tachycardia, or other treatment emergent cardiac arrhythmias after administration of sugammadex. Approximately 331 subjects will be enrolled (randomized) across multiple global sites.

The trial will consist of four visits: screening visit (V1), peri-anesthetic visit (V2), post-anesthetic visit (V3), and a follow-up safety contact (V4). The screening and peri-anesthetic visits may occur on the same day (see Section 7.1.5.2). Subjects will be in the trial for approximately 30 days.

Subjects will be stratified by ASA Physical Status Class by an Interactive Voice Response System (IVRS), to facilitate enrollment of the target number of randomized subjects into the ASA Class 4 stratum (approximately 25% of the overall planned study sample).

Subjects will also be stratified by neuromuscular blocking agent (NMBA), rocuronium or vecuronium. A total of ~30% of the total number of randomized subjects will be in the vecuronium stratum (i.e., ~100 subjects). Investigators will be permitted to select the NMBA, for use as appropriate for the type of surgery provided that both NMBA strata remain open.

Subjects will be randomized to depth of NMB (moderate or deep) and reversal agent (sugammadex or neostigmine + glycopyrrolate).

Within the rocuronium stratum, subjects will be randomized to one of four treatment groups in a 2:1:2:2 ratio as follows (N=~231):

1. Moderate block and reversal with sugammadex 2 mg/kg
2. Moderate block and reversal with neostigmine(50 µg/kg up to 5 mg maximum dose) + glycopyrrolate (10 µg/kg up to 1 mg maximum dose)
3. Deep block and reversal with sugammadex 4 mg/kg
4. Deep block and reversal with sugammadex 16 mg/kg

Within the vecuronium stratum, subjects will be randomized to one of three treatment groups in a 2:1:2 ratio as follows (N=~100):

1. Moderate block and reversal with sugammadex 2 mg/kg
2. Moderate block and reversal with neostigmine (50 µg/kg up to 5 mg maximum dose) + glycopyrrolate (10 µg/kg up to 1 mg maximum dose)
3. Deep block and reversal with sugammadex 4 mg/kg

Depending on treatment assignment, subjects should be maintained, according to standard medical practice, in either moderate or deep NMB intraoperatively until the time of reversal:

- Subjects randomized to moderate block should be maintained, according to standard medical practice, in moderate block until the time of reversal. Either sugammadex 2 mg/kg or neostigmine + glycopyrrolate will be given after the last dose of administered NMBA and within 5 minutes of detection of reappearance of second twitch (T2) with a lower limit of train of four (TOF) count 1 and upper limit of TOF count of 4.
- Subjects randomized to deep block should be maintained, according to standard medical practice, in deep block until the time of reversal. Either sugammadex 4 mg/kg or 16 mg/kg will be given after the last dose of administered NMBA and within 5 minutes of detection of post-tetanic counts (PTC) ≥1 and a TOF count of 0.

Safety endpoints include the incidence of treatment emergent sinus bradycardia, treatment emergent sinus tachycardia, or other treatment emergent cardiac arrhythmias after administration of sugammadex or neostigmine. For the purpose of this study:

- **Treatment emergent sinus bradycardia** is defined as meeting all of the following:
    - a heart rate <60 bpm
    - a heart rate decreased more than 20% compared to the subject's baseline heart rate value*
    - is sustained for at least 1 minute after administration of study medication.
- **Treatment emergent sinus tachycardia** is defined as meeting all of the following:
    - a heart rate ≥100 bpm
    - a heart rate increased more than 20% compared to the subject's baseline heart rate value*
    - is sustained for at least 1 minute after administration of study medication.
- **Other treatment emergent cardiac arrhythmias** are defined as meeting all of the following:
    - new or worsening arrhythmias (e.g., atrial fibrillation, atrial tachycardia, ventricular fibrillation, or ventricular tachycardia),
    - is sustained for at least 1 minute after administration of study medication.

*Baseline heart rate is defined as the heart rate taken after the last dose of NMBA is administered and (approximately 5 minutes) prior to administration of study medication.

Safety assessments, including AEs, serious adverse events (SAEs), and events of clinical interest (ECIs), will be recorded during the trial as noted in the Trial Flow Chart in Section 6.0. Any perioperative events that occur in the operating room (OR) or post-anesthesia care unit (PACU) will be recorded by study personnel that are present.

To ensure timely reporting and comprehensive data collection, the following types of events are pre-specified as ECIs:

- Clinically relevant arrhythmias, inclusive of bradycardia and tachycardia, defined as events necessitating intervention, as determined by investigator judgment.
- Hypersensitivity and/or anaphylaxis
    - Potential cases of hypersensitivity and/or anaphylaxis will be adjudicated by an external expert Adjudication Committee.

For this study, the anesthesiologist (or comparable professionally qualified individual, such as certified nurse anesthetist), who is present during the operation and administers the study medication, will be blinded to the reversal agent in the moderate block arms. While sugammadex for the deep block arm (as for the moderate block arm) will be provided to the anesthesiologist in masked syringes, OR staff aware of the depth of block will know the study medication as there is no approved comparator for reversal of deep block. However, the dose of sugammadex in the rocuronium deep block groups will remain blinded.

This study will have a blinded safety assessor (BSA). For any given individual subject, the anesthesiologist performing study related procedures during the surgery and the BSA must be two separate individuals, and their roles and responsibilities must not overlap. The BSA, for any given individual subject, will not be present during the operation and will not administer study medication. The BSA will be blinded to study medication assignment, depth of NMB, and drug preparation records. Of note, a BSA may, for any other subject, fill other study roles as appropriate for his/her qualifications. Note: if the BSA is not a physician, a blinded physician (MD, DO) will be responsible for completing the causality assessment for all adverse events, including any perioperative adverse events.

Specific procedures to be performed during the trial, as well as their prescribed times and associated visit windows, are outlined in the Trial Flow Chart - Section 6.0. Details of each procedure are provided in Section 7.0 – Trial Procedures.

2.2 Trial Diagram

The trial design is depicted in Figure 1.

Randomization Scheme

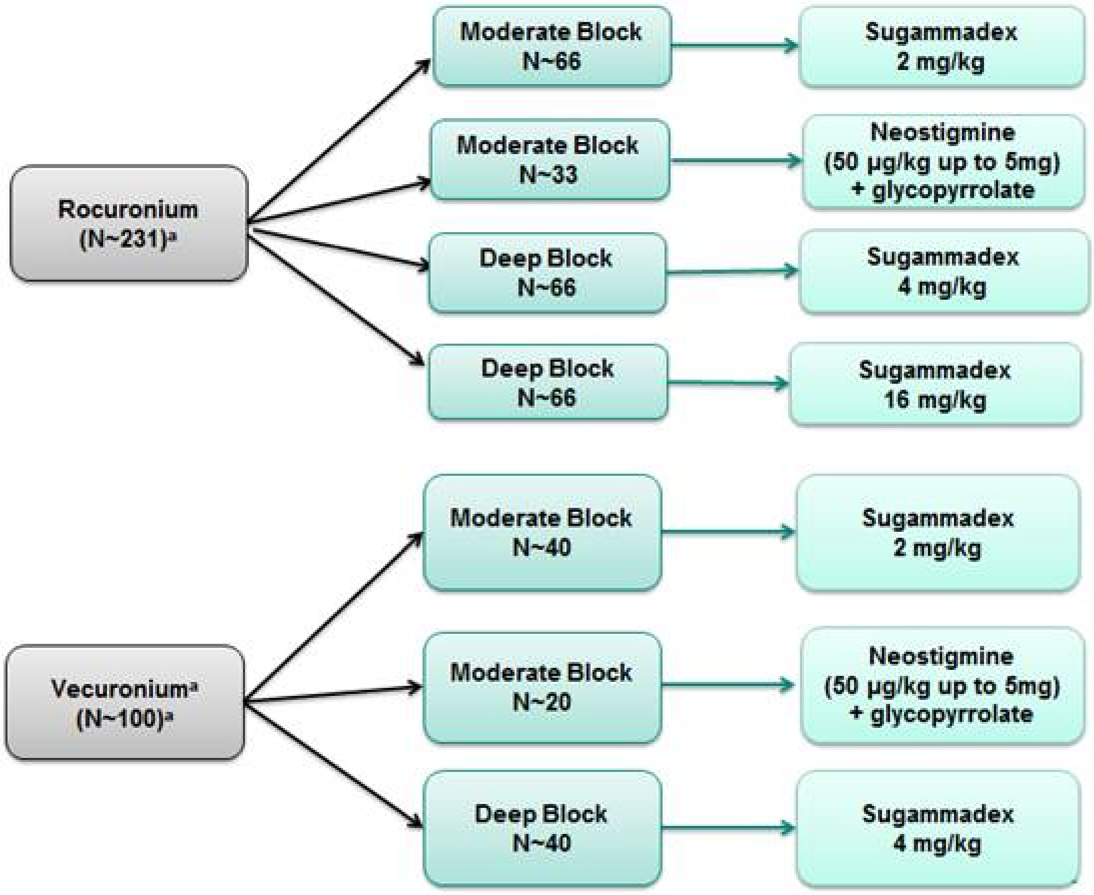

Subjects will be stratified by ASA Physical Class by an Interactive Voice Response System (IVRS), which will ensure the target number of randomized subjects are enrolled in the ASA Class 4 stratum (approximately 25% of the overall planned study sample).

[a] Subjects will also be stratified by neuromuscular blocking agent (NMBA), rocuronium or vecuronium. A total of ~30% of the total number of randomized subjects will be in the vecuronium stratum (ie, ~100 subjects).

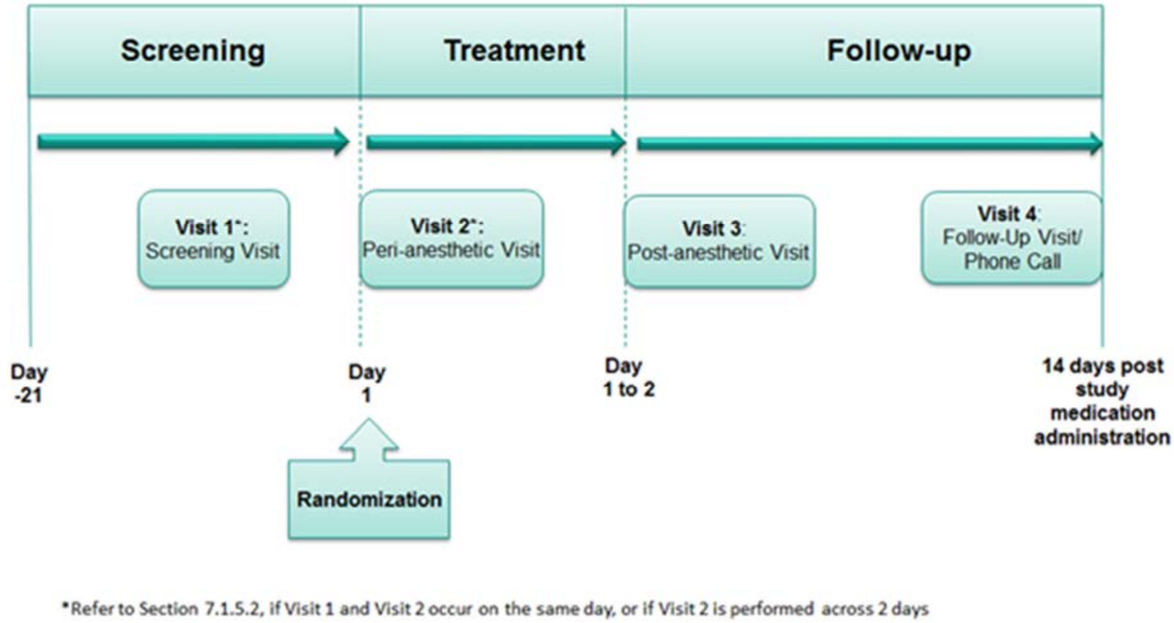

Figure 1 Trial Design

3.0 OBJECTIVE(S) & HYPOTHESIS(ES)

3.1 Primary Objective(s) & Hypothesis(es)

1) **Objective**: To characterize the incidence of treatment emergent sinus bradycardia, treatment emergent sinus tachycardia, or other treatment emergent cardiac arrhythmias after administration of sugammadex in a population of ASA Class 3 and 4 subjects in a surgical setting.
2) **Objective**: To evaluate the general safety of sugammadex in a population of ASA Class 3 and 4 subjects in a surgical setting.

3.2 Secondary Objective(s) & Hypothesis(es)

There are no secondary objectives (or hypotheses) for this trial.

4.0 BACKGROUND & RATIONALE

4.1 Background

Refer to the Investigator's Brochure (IB)/approved labeling for detailed background information on MK-8616.

4.1.1 Pharmaceutical and Therapeutic Background

Sugammadex sodium, herein referred to as sugammadex is a modified gamma-cyclodextrin administered at the end of surgical procedures to reverse paralysis induced by the steroidal NMBAs rocuronium and vecuronium. Sugammadex accomplishes this reversal of neuromuscular blockade (NMB) through formation of high affinity sugammadex: NMBA complexes. Given the very high binding affinity and low dissociation rate of the complex, the bound NMBAs can no longer act at the neuromuscular junction, thereby restoring muscle function. The complex is then renally eliminated. Since this mechanism of action does not involve direct interaction with cholinergic systems, it circumvents undesired side effects associated with acetylcholinesterase inhibitors. Furthermore, sugammadex does not require the presence of neuromuscular activity before administration, and is therefore effective in reversal of both moderate and deep levels of NMB.

Sugammadex has been extensively studied in 58 clinical trials with a total of 5999 exposures to IV sugammadex in 4453 unique individuals, establishing a well characterized safety and efficacy profile in adults. It is currently approved in more than 83 countries and marketed in more than 50 countries with more than an estimated 30 million exposures worldwide at the time of authoring of this document.

4.2 Rationale

4.2.1 Rationale for the Trial and Selected Subject Population

In adults, most demographic factors, such as age, body weight, or other factors related to the medical history or surgical procedures have not been found to affect sugammadex efficacy or safety. While sugammadex exposure is increased in the setting of renal impairment, the safety and efficacy of sugammadex has been shown to not be impacted by mild to moderate levels of renal impairment.

In the combined clinical safety database, evaluation of cardiac arrhythmia AEs did not show increases in the incidence of arrhythmia-related AEs after sugammadex treatment as compared to placebo. Rates of reporting of arrhythmias after use of sugammadex in patients in the postmarketing environment were generally no higher than and in most cases were lower than the reported rates of peri/post-operative arrhythmias in the literature [1][2][3]. The only exception was rare observations of marked bradycardia in patients (following sugammadex administration for the reversal of NMBAs), which were readily detectable in the well-monitored post-operative setting, and typically responded well to usual anticholinergic therapy.

The risk of bradycardia related to sugammadex appears to be lower than that associated with the alternative usual treatment neostigmine, which, as a cholinesterase inhibitor, has pronounced pro-cholinergic effects. However, given this risk patients should be closely monitored for hemodynamic changes during and after reversal of neuromuscular blockade, and treatment with anti-cholinergic agents such as atropine should be administered if clinically significant bradycardia is observed.

In terms of ASA class, the pharmacokinetic (PK), efficacy, and safety results were generally consistent across ASA class groups. However, the number of subjects in Phase 3 studies who met ASA class 3 or 4 was small. Since ASA class 3 or 4 subjects may have increased cardiac risk given their comorbidity and status, the current trial aims to demonstrate that sugammadex is generally safe for reversing rocuronium or vecuronium-induced NMB (both moderate and deep block) in subjects undergoing surgery who meet the ASA class 3 or 4 criteria.

4.2.2 Rationale for Dose Selection/Regimen

Depending on the selected NMBA and the randomly assigned depth of NMB (moderate or deep), a sugammadex dose of either 2 mg/kg, 4 mg/kg, or 16 mg/kg, based on actual body weight, will be administered as an intravenous (IV) bolus dose to reverse NMB. The 2 mg/kg and 4mg/kg dose has been established for reversal of moderate or deep block, respectively. The 16 mg/kg sugammadex dose is indicated for immediate reversal of rocuronium 1.2 mg/kg.

The 16 mg/kg dose has been evaluated as part of the extensive clinical development program for sugammadex in subjects receiving both NMBA and general anesthesia with a total of 98 subjects receiving the 16 mg/kg dose. In placebo-controlled trials of subjects treated with NMBA and general anesthesia, the incidence of adverse events (AE) reported in those receiving sugammadex compared favorably with those receiving placebo. In the Pooled Phase 1-3 group, the following drug-related AEs as determined by the investigator associated with exposure to 16 mg/kg doses of sugammadex were reported at an incidence of ≥2%: anaesthetic complication (n=2, 2%), procedural complication (n=3, 3%), procedural hypertension (n=2,2%), nausea (n=3, 3%), bradycardia (n=2, 2%), and tachycardia (n=4, 4%). There were no other drug-related AEs except nausea (2%) that occurred at ≥2% at 2 or 4 mg/kg in the Pooled Phase 1-3 trials.

Hypersensitivity reactions, including anaphylaxis, have occurred in both premarketing clinical trials and in post-marketing spontaneous reports. In a randomized, double-blind placebo-controlled study the incidence of drug hypersensitivity reactions in healthy subjects not receiving anesthesia who received up to 3 doses of placebo (N=76), sugammadex 4 mg/kg (N=151) or sugammadex 16 mg/kg (N=148), separated by approximately 5 weeks was examined. The frequency of anaphylaxis in the sample of 299 healthy volunteers was 0.3% (one incidence in 16 mg/kg group, none in 4 mg/kg or placebo group). These reactions varied from isolated skin reactions to serious systemic reactions (i.e., anaphylaxis, anaphylactic shock) and have occurred in patients with no prior exposure to sugammadex.

In the Pooled Phase 1-3 trials, the overall incidence of adverse events was balanced between groups, without evidence for a pro-arrhythmic effect of sugammadex. In the Broad and Narrow Standardized MedDRA Query (SMQ) for ‘Cardiac Arrhythmias’ there was a comparable incidence for total sugammadex (broad 4.6%, narrow 1%) and for placebo (broad 4.0%, narrow 1.8%). However, the incidence of bradyarrhythmias (including conduction defects and disorders of sinus node function) was potentially dose-dependent (2%, 2.3%, 1.3%, and 4.1% for placebo, 2 mg/kg, 4 mg/kg, and 16 mg/kg, respectively). Therefore, study of all three indicated doses of sugammadex is essential to further characterize the safety profile of sugammadex in ASA 3 and 4 subjects. Because it is not feasible to study the

safety of the 16 mg/kg dose in the emergent setting to reverse an intubating dose of rocuronium, for this study 16 mg/kg will be administered for reversal of deep block to enable characterization of its risk for arrhythmias.

4.2.2.1 Rationale for the Use of Comparator

For reversal of moderate NMB, neostigmine is selected as an active comparator because it is the most frequently used acetylcholinesterase inhibitor indicated for reversal of moderate block. As per current prescribing information, neostigmine will be co-administered intravenously with glycopyrrolate to counter the anticipated muscarinic effects of neostigmine. Neostigmine will be administered at the recommended dose of 50 µg/kg, up to a maximum dose of 5 mg, and glycopyrrolate will be administered at a dose of 10 µg/kg, up to a maximum dose of 1 mg. Hereafter, reference to the administration of "neostigmine" herein or in other study documents pertains to the administration of neostigmine + glycopyrrolate as noted.

Because sugammadex is the only reversal agent indicated for reversal of deep NMB, no deep block comparator is available in this trial. However, the neostigmine group will provide a standard-of-care comparison for the sugammadex 4 and 16 mg/kg groups with respect to safety.

4.2.3 Rationale for Endpoints

4.2.3.1 Efficacy Endpoints

Not applicable as this is a safety trial.

4.2.3.2 Safety Endpoints

The primary safety endpoint in this trial is to characterize the incidence of treatment emergent sinus bradycardia, treatment emergent sinus tachycardia, or other treatment emergent cardiac arrhythmias after administration of sugammadex among ASA Class 3 and 4 subjects. Other safety events (e.g., recurrence of NMB or respiratory events) and tolerability of sugammadex will be continuously assessed throughout the trial via standard AE reporting.

Thorough assessment for potential treatment related arrhythmias, inclusive of treatment emergent sinus bradycardia, and treatment emergent sinus tachycardia, is planned. Continuous ECG monitoring will be performed, beginning at least 5 minutes before, during, and for at least 30 minutes after administration of study medication to facilitate arrhythmia assessment.

For the purpose of this study:

- **Treatment emergent sinus bradycardia** is defined as meeting <u>all</u> of the following:
  - a heart rate <60 bpm
  - a heart rate decreased more than 20% compared to the subject's baseline heart rate value*
  - is sustained for at least 1 minute after administration of study medication.

- **Treatment emergent sinus tachycardia** is defined as meeting all of the following:
  - a heart rate ≥100 bpm
  - a heart rate increased more than 20% compared to the subject's baseline heart rate value*
  - is sustained for at least 1 minute after administration of study medication.
- **Other treatment emergent cardiac arrhythmias** are defined as meeting all of the following:
  - new or worsening arrhythmias (e.g., atrial fibrillation, atrial tachycardia, ventricular fibrillation, or ventricular tachycardia)
  - is sustained for at least 1 minute after administration of study medication.

*Baseline heart rate is defined as the heart rate taken after the last dose of NMBA is administered and (approximately 5 minutes) prior to administration of study medication.

To ensure timely reporting and comprehensive data collection regarding ECIs, the following types of events are pre-specified as ECIs:

- **Clinically relevant sinus bradycardia** is defined as any bradycardia event necessitating intervention, as determined by investigator judgment
- **Clinically relevant sinus tachycardia** is defined as any tachycardia event necessitating intervention, as determined by investigator judgment
- **Other clinically relevant cardiac arrhythmias** are defined as any arrhythmia event necessitating intervention, as determined by investigator judgment.
- **Hypersensitivity and/or anaphylaxis**

  Events of hypersensitivity and anaphylaxis (defined by Sampson et al. [4]) will also be adjudicated by an independent external Adjudication Committee.

Any perioperative events that occur in the OR or PACU will be recorded by study personnel that are present.

Lastly, standard safety assessments (e.g., physical examination, vital signs including heart rate and blood pressure) will be recorded at specified time points (see Trial Flow Chart – Section 6.0).

4.2.3.3 Future Biomedical Research

The Sponsor will conduct Future Biomedical Research on DNA specimens consented for future biomedical research during this clinical trial.

Such research is for biomarker testing to address emergent questions not described elsewhere in the protocol (as part of the main trial) and will only be conducted on specimens from appropriately consented subjects. The objective of collecting/retaining specimens for Future Biomedical Research is to explore and identify biomarkers that inform the scientific understanding of diseases and/or their therapeutic treatments. The overarching goal is to use such information to develop safer, more effective drugs/vaccines, and/or to ensure that subjects receive the correct dose of the correct drug/vaccine at the correct time. The details

of this Future Biomedical Research sub-trial are presented in Section 12.2 – Collection and Management of Specimens for Future Biomedical Research.

4.3 Benefit/Risk

Subjects in clinical trials generally cannot expect to receive direct benefit from treatment during participation, as clinical trials are designed to provide information about the safety and effectiveness of an investigational medicine.

Sugammadex has a positive benefit-risk profile and is well tolerated in the approved indications as described in the Investigator's Brochure. It has specifically been shown to be superior (faster and effective in a higher proportion of treated subjects) to both placebo and neostigmine for reversal of moderate and deep NMB. Reversal of deep NMB is a unique benefit of sugammadex unavailable with currently available treatments. From a risk perspective, sugammadex has been shown to be generally safe and well tolerated. The use of sugammadex at recommended doses is associated with a low risk of residual NMB or recurrence of NMB compared with current treatment. The clinical trial experience with sugammadex (with 5999 subject IV exposures) is consistent with and supported by post-marketing data obtained in real world use in clinical practice (with more than an estimated 30 million patients exposed in more than 50 countries).

Additional details regarding specific benefits and risks for subjects participating in this clinical trial may be found in the accompanying IB and Informed Consent documents.

5.0 METHODOLOGY

5.1 Entry Criteria

5.1.1 Diagnosis/Condition for Entry into the Trial

Male/female subjects aged 18 years or older who meet the American Society of Anesthesiologists Physical Status Class of 3 or 4 will be enrolled in this trial.

5.1.2 Subject Inclusion Criteria

In order to be eligible for participation in this trial, the subject must:

1. Have a body mass index (BMI) < 40 kg/m^2.
2. Be categorized as ASA Physical Status Class 3 or 4, as determined by the Investigator [5]. Note that subject should meet criteria for ASA Class 3 or 4, independent of the BMI $\geq$40 kg/m^2 criterion.
3. Have a planned surgical procedure that requires neuromuscular block with either rocuronium or vecuronium.
4. Have a planned surgical procedure (e.g., gastrointestinal [GI], urologic, or laparoscopic procedures) that in the opinion of the investigator does not preclude maintenance of moderate or deep depth of NMB throughout the case (maintained by re-dosing or continuous infusion).

5. If female, who is not of reproductive potential be one of the following: (1) postmenopausal (defined as at least 12 months with no menses in women ≥45 years of age); (2) has had a hysterectomy and/or bilateral oophorectomy, bilateral salpingectomy, or bilateral tubal ligation/occlusion at least 6 weeks prior to screening; (3) has a congenital or acquired condition that prevents childbearing; or (4) is undergoing surgical sterilization as the planned surgical procedure associated with participation in this study (e.g., hysterectomy or tubal ligation).

6. If female, who is sexually active and of child-bearing potential, agrees to use a medically accepted method of contraception through seven days after receiving protocol-specified medication. Medically accepted methods of contraception include condoms (male or female) with a spermicidal agent, diaphragm, or cervical cap with spermicide, medically prescribed intrauterine device (IUD), inert or copper-containing IUD, surgical sterilization (e.g., hysterectomy or tubal ligation). Abstinence (relative to heterosexual activity) can be used as the sole method of contraception if it is consistently employed as the subject's preferred and usual lifestyle and if considered acceptable by local regulatory agencies and ERCs/IRBs. Periodic abstinence (e.g., calendar, ovulation, sympto-thermal, post-ovulation methods, etc.) and withdrawal are not acceptable methods of contraception.

   If a contraceptive method listed above is restricted by local regulations/guidelines, then it does not qualify as an acceptable method of contraception for subjects participating at sites in this country/region.

7. Be able to provide (or the subject's legally authorized representative, in accordance with local requirements), written informed consent for the trial. The subject or legally authorized representative may also provide consent for Future Biomedical Research. However, the subject may participate in the main trial without participating in Future Biomedical Research.

5.1.3 Subject Exclusion Criteria

The subject must be excluded from participating in the trial if the subject:

1. Has a pacemaker or automatic implantable cardioverter-defibrillator (AICD) that precludes the assessment of bradycardia or arrhythmias.
2. Has a medical condition or surgical procedure that precludes reversal of neuromuscular block at the end of surgery.
3. Has a neuromuscular disorder(s) that may affect neuromuscular block and/or trial assessments.

4. Is dialysis-dependent or has severe renal insufficiency.

   Note: If dialysis or severe renal insufficiency is not known or confirmed in the subject's medical history, a creatinine clearance value should be calculated based upon serum creatinine values obtained from local labs within the past 2 weeks prior to Visit 1 or local labs performed between V1 and V2 (e.g., pre-operative testing). Severe renal insufficiency is defined as an estimated creatinine clearance of < 30 mL/min using the formulas below.

   Cockcroft-Gault formula:

   For Males: $$\text{Creatinine Clearance} = \frac{(140\text{-age [years]}) \times \text{weight (kg)}}{\text{serum creatinine (mg/dL)} \times 72}$$

   For Females: $$\text{Creatinine Clearance} = \frac{0.85 \times (140\text{-age [years]}) \times \text{weight (kg)}}{\text{serum creatinine (mg/dL)} \times 72}$$

5. Has or is suspected of having a personal history or family history (parents, grandparents, or siblings) of malignant hyperthermia.
6. Has or is suspected of having an allergy (e.g., hypersensitivity and/or anaphylactic reaction) to study treatments or its/their excipients, to opioids/opiates, muscle relaxants or their excipients, or other medication(s) used during general anesthesia.
7. Has received or is planned to receive toremifene within 24 hours before or within 24 hours after study medication administration.
8. Has any condition that would contraindicate the administration of study medication.
9. Is pregnant, is attempting to become pregnant, or is lactating.
10. Has any clinically significant condition or situation (e.g., anatomical malformation that complicates intubation) other than the condition being studied that, in the opinion of the investigator, would interfere with the trial evaluations or optimal participation in the trial.
11. Is currently participating in or has participated in an interventional clinical trial (including any other current or ongoing trial with a sugammadex treatment arm) with an investigational compound or device within 30 days of signing the informed consent form of this current trial.
12. Is or has an immediate family member (e.g., spouse, parent/legal guardian, sibling or child) who is investigational site or sponsor staff directly involved with this trial.

5.2 Trial Treatment(s)

The treatments to be used in this trial are outlined below in Table 1.

Table 1 Trial Treatments

Drug	Dose/Potency	Dose Frequency	Route of Administration	Treatment Period	Use
Sugammadex	2 mg/kg	Single dose	IV	Peri-anesthetic period	Experimental
Sugammadex	4 mg/kg	Single dose	IV	Peri-anesthetic period	Experimental
Sugammadex	16 mg/kg	Single dose	IV	Peri-anesthetic period	Experimental
Neostigmine<br>+<br>Glycopyrrolate	50 µg/kg (up to 5 mg maximum dose)<br>10 µg/kg (up to 1 mg maximum dose)	Single dose	IV	Peri-anesthetic period	Comparator/ Standard of Care

Trial Treatment should begin within 1 day of randomization. Randomization in IVRS may occur on the same day as a subject's scheduled surgery or one day in advance of the scheduled surgery. Sites should contact the Sponsor if a subject's surgery is postponed for more than one day from the date the subject is randomized in IVRS (e.g., surgery postponed due to subject illness). Trial status of subjects in these situations will be handled on a case by case basis in consultation with the Sponsor. See Section 7.1.5.2.

The investigator shall take responsibility for and shall take all steps to maintain appropriate records and ensure appropriate supply, storage, handling, distribution and usage of trial treatments in accordance with the protocol and any applicable laws and regulations.

5.2.1 Dose Selection

5.2.1.1 Dose Selection (Preparation)

The rationale for selection of doses to be used in this trial is provided in Section 4.0 – Background & Rationale.

Sugammadex, neostigmine and glycopyrrolate will be prepared by an unblinded pharmacist (or delegate) and supplied to OR staff in masked syringes for administration to study subjects.

The volume of study medication drawn into the syringes will be normalized between treatment groups by adding saline to allow for blinding of differences in volume and

concentrations for assigned doses. Instructions for normalizing the volumes are included in a separate Study Drug Manual.

5.2.2 Timing of Dose Administration

Trial treatment should begin within 1 day of randomization in IVRS.

The total duration of administration for all study medication syringes (Dose 1 and Dose 2 syringe(s) as outlined in the Study Drug Manual) should not exceed a total of 20 seconds. The syringes should be administered as a bolus injection into a fast running intravenous line, on the day of surgery.

For subjects randomized to moderate block, sugammadex or neostigmine will be given after the last dose of administered NMBA and within 5 minutes of detection of reappearance of T2 (with a lower limit of TOF count 1 and upper limit of TOF count of 4).

For subjects randomized to deep block, sugammadex will be given after the last dose of administered NMBA and within 5 minutes of detection of PTC $\geq$1 and a TOF count of 0.

5.2.3 Trial Blinding

Study Medication Blinding

With the exception of designated individuals (e.g., unblinded Clinical Research Associates [CRAs]), all other Sponsor study team personnel will be blinded to study medication assignments for randomized subjects.

The site **pharmacist (or delegate)** will be **unblinded** to study medication assignments in order to prepare study medication. Study medication will be provided to site staff in the OR (anesthesiologist) in masked syringes to ensure that the contents of the syringes will not be revealed.

Site Personnel Roles and Blinding

The anesthesiologist (or comparable professionally qualified individual, such as certified nurse anesthetist), and other OR staff will be blinded to the reversal agent in the moderate block arms. While sugammadex for the deep block arm will be provided to the OR (anesthesiologist) in masked syringes, OR staff aware of the depth of block will know the study medication as there is no approved comparator for reversal of deep block. However, the dose of sugammadex in the rocuronium deep block groups will remain blinded.

The **blinded safety assessor (BSA)** will be an appropriately qualified health care professional (e.g., a licensed physician, nurse practitioner, physician assistant, or certified registered nurse anesthetist [or comparable professional qualification in countries outside of the United States]) with training and experience in anesthesia or post-anesthesia care.

- The **BSA** for any given individual subject will be blinded to:
  - Study medication assignment
  - The depth of NMB
  - Drug preparation records

- The **BSA** for any given individual subject will:
  - o Not be present during the operation and will not administer study medication
  - o Conduct the required procedures (see Section 6.0 Trial Flow Chart) at the post-anesthetic safety visit (Visit 3)
  - o Complete the causality assessment for all adverse events, including any perioperative adverse events. Note: if the BSA is not a physician, a blinded physician (MD, DO), not present during the surgical procedure, reversal dosing, or recovery for a given subject, will be responsible for completing the causality assessment for all adverse events.
  - o Be required to sign a statement for each subject (V4 or discontinuation) confirming that the blind was maintained (treatment group, depth of block, drug preparation records).
- Of note, a **BSA** may, for any other subject, fill other study roles as appropriate for his/her qualifications.

The anesthesiologist performing study-related procedures during surgery and the BSA must be 2 separate individuals, and their roles and responsibilities must not overlap for any given individual subject.

Any inadvertent unblinding of the BSA will be documented.

See Section 7.1.4.1.3, Blinding/Unblinding, for a description of the method of unblinding a subject during the trial, should such action be warranted.

5.3 Randomization

Randomization will occur centrally using the IVRS.

Within the rocuronium stratum, subjects will be randomized to 1 of 4 treatment groups as indicated in the Trial Diagram (Figure 1 in Section 2.2).

Subjects will be assigned randomly on Day 1 in a 2:1:2:2 ratio:

1. Moderate block and reversal with sugammadex 2 mg/kg
2. Moderate block and reversal with neostigmine 50 µg/kg + glycopyrrolate 10 µg/kg
3. Deep block and reversal with sugammadex 4 mg/kg
4. Deep block and reversal with sugammadex 16 mg/kg

Within the vecuronium stratum, subjects will be randomized to one of three treatment groups as indicated in the Trial Diagram (Figure 1 in Section 2.2).

Subjects will be assigned randomly on Day 1 in a 2:1:2 ratio:

1. Moderate block and reversal with sugammadex 2 mg/kg
2. Moderate block and reversal with neostigmine 50 µg/kg + glycopyrrolate 10 µg/kg
3. Deep block and reversal with sugammadex 4 mg/kg

5.4 Stratification

Treatment allocation/randomization will be stratified according to the following factors:

- NMBA (rocuronium or vecuronium)
- ASA Physical Status Class 3 or 4

A total of ~30% of the total number of randomized subjects will be in the vecuronium stratum (i.e., ~100 subjects).

Subjects will be stratified by ASA Physical Status Class by an IVRS system, which will ensure the target number of randomized subjects are enrolled in the ASA Class 4 stratum (approximately 25% of the overall planned study sample).

5.5 Concomitant Medications/Vaccinations (Allowed & Prohibited)

Medications or vaccinations specifically prohibited in the exclusion criteria are not allowed during the ongoing trial. If there is a clinical indication for any medication or vaccination specifically prohibited during the trial, the investigator should discuss this with the Sponsor Clinical Director. The final decision on any supportive therapy or vaccination rests with the investigator and/or the subject's primary physician.

Rocuronium or vecuronium are concomitant medications to be used per label as adjunct to general anesthesia. Besides rocuronium and vecuronium, a subject must not be administered any other NMBAs during the trial, including:

- Other steroidal NMBAs such as pancuronium
- Nonsteroidal NMBAs such as succinylcholine or benzylisoquinolinium compound (e.g., cisatracurium)*

Toremifene use within 24 hours before or within 24 hours after study medication administration is prohibited.

*Except in the circumstance that renewed muscle relaxation is needed after administration of study medication, in which case a non-steroidal NMBA should be administered.

5.6 Rescue Medications & Supportive Care

No rescue or supportive medications are specified to be used in this trial.

5.7 Diet/Activity/Other Considerations

There are no dietary or activity restrictions required for this protocol.

5.8 Subject Withdrawal from the Trial

A subject must be withdrawn from the trial if the subject or subject’s legally acceptable representative withdraws consent from the trial.

If a subject withdraws from the trial, they will not receive treatment or be followed at scheduled protocol visits.

Specific details regarding procedures to be performed at the time of withdrawal from the trial including the procedures to be performed should a subject fail to return for scheduled visits and/or if the study site is unable to contact the subject, as well as specific details regarding withdrawal from Future Biomedical Research are outlined in Section 7.1.4 – Other Procedures.

5.9 Subject Replacement Strategy

A subject who discontinues from the trial will not be replaced.

5.10 Beginning and End of the Trial

The overall trial begins when the first subject signs the informed consent form. The overall trial ends when the last subject completes the last study-related phone-call or visit, discontinues from the trial or is lost to follow-up (i.e. the subject is unable to be contacted by the investigator).

5.11 Clinical Criteria for Early Trial Termination

There are no pre-specified criteria for terminating the trial early.

6.0 TRIAL FLOW CHART

Trial Period:	Screening	Treatment	Follow-up	
Visit Number/Title:	**1**<br>Screening	**2**<br>Peri-anesthetic Visit	**3**<br>Post-anesthetic Visit [a]	**4**<br>Follow-up Safety Contact[b]
Scheduled Day:	**Between Days -21 to 1**	**Day 1**<br>(Day of Surgery)	**Days 1 to 2**<br>(4 to 36 hours post study medication administration)	14 days post study medication administration
Scheduling Window:	-21 to Day 1	±0 days	See footnote “c”	+2 days
	**Visit 1 and Visit 2 may occur on same day. Visit 2 may occur across 2 days. Refer to Section 7.1.5.2.**			
**Administrative Procedures**				
Informed Consent	X			
Informed Consent for Future Biomedical Research	X			
Inclusion/Exclusion Criteria	X	X		
Subject Identification Card	X	X[d]		
Medical History	X			
Concomitant Medication Review	X	X	X	X
Screening Number Assignment	X			
Randomization[e]		X		
**Clinical Procedures/Assessments**				
Physical Examination	X		X [a]	
Height	X			
Weight and BMI calculation	X	X		
12-Lead Electrocardiogram	X			

Trial Period:	Screening	Treatment	Follow-up	
Visit Number/Title:	**1** Screening	**2** Peri-anesthetic Visit	**3** Post-anesthetic Visit [a]	**4** Follow-up Safety Contact[b]
Scheduled Day:	**Between Days -21 to 1**	**Day 1** (Day of Surgery)	**Days 1 to 2** (4 to 36 hours post study medication administration)	14 days post study medication administration
Scheduling Window:	-21 to Day 1	±0 days	See footnote "c"	+2 days
	**Visit 1 and Visit 2 may occur on same day. Visit 2 may occur across 2 days. Refer to Section 7.1.5.2.**			
Continuous ECG Monitoring		X[f]		
Vital Signs (heart rate, respiratory rate, O_2 saturation, blood pressure, temperature)	X	X[g]	X	
Administration of NMBA		X		
Administration of Study Medication		X		
Neuromuscular Monitoring[h]		X		
Adverse Events/ ECI Monitoring	X	X	X [a]	X
**Laboratory Procedures/Assessments**				
Hematology[i]		X	X	
Chemistry[i]		X	X	
Pregnancy Test[j]	X	X[j]		
Blood (DNA) for Future Biomedical Research[k]		X		

Trial Period:	Screening	Treatment	Follow-up	
Visit Number/Title:	**1**<br>Screening	**2**<br>Peri-anesthetic Visit	**3**<br>Post-anesthetic Visit [a]	**4**<br>Follow-up Safety Contact[b]
Scheduled Day:	**Between Days -21 to 1**	**Day 1**<br>(Day of Surgery)	**Days 1 to 2**<br>(4 to 36 hours post study medication administration)	14 days post study medication administration
Scheduling Window:	-21 to Day 1	±0 days	See footnote "c"	+2 days
	**Visit 1 and Visit 2 may occur on same day.**<br>**Visit 2 may occur across 2 days.**<br>**Refer to Section 7.1.5.2.**			

[a] The physical exam and adverse event review must be conducted by the BSA at V3. For any given individual subject, the anesthesiologist performing study-related procedures during the surgery and the BSA must be two separate individuals and their roles and responsibilities will not overlap.
[b] Should occur via phone, however, may be performed in person if the subject is still in the hospital.
[c] Post-anesthetic visit will be completed a minimum of 4 hours or up to a maximum of 36 hours after administration of study medication or discharge, whichever occurs first.
[d] Randomization number to be added at Visit 2.
[e] Trial treatment should begin within 1 day of randomization. Randomization in IVRS may occur on the same day or one day in advance of the subject's scheduled surgery (except when V1 and V2 occur on the same day). Sites should contact the Sponsor if a subject's surgery is postponed for more than 1 day from the date the subject is randomized in IVRS (e.g., surgery postponed due to subject illness). Trial status of subjects in these situations will be handled on a case by case basis in consultation with the Sponsor.
[f] To occur at least 5 minutes before, during, and for 30 minutes following administration of study medication.
[g] To be performed pre-administration of NMBA, approximately 5 minutes prior to study medication administration, and at 2, 5, 10, and 30 minutes following administration of study medication.
[h] Neuromuscular monitoring is to be done using locally available NMTM (e.g., objective NMTM or subjective NMTM using a PNS).
[i] Laboratory samples will be sent to a central laboratory for analysis. Baseline safety labs should be obtained pre-NMBA administration and post-op safety labs are to be drawn 4-6 hours post-administration of sugammadex/neostigmine.
[j] For females of childbearing potential, a urine or serum β-hCG pregnancy test is required within 24 hours prior to surgery. A serum β-hCG pregnancy test (performed locally) is required if the urine pregnancy test is positive, unless local requirements require otherwise. Note that if the pregnancy test is done within 24 hours prior to surgery at Visit 1, it does not have to be repeated at Visit 2.
[k] Informed consent for future biomedical research samples must be obtained to collect the DNA sample. The blood (DNA) for future biomedical research sample should be taken on Day 1 at the same time as other blood collections. If the sample cannot be taken at this visit, it can be taken at the next scheduled blood draw.

Abbreviations: BMI = Body mass index; NMBA = Neuromuscular blocking agent; ECI = Events of clinical interest; β-hCG = Serum beta human chorionic gonadotropin; DNA = Deoxyribonucleic acid; BSA = Blinded Safety Assessor

7.0 TRIAL PROCEDURES

7.1 Trial Procedures

The Trial Flow Chart - Section 6.0 summarizes the trial procedures to be performed at each visit. Individual trial procedures are described in detail below. It may be necessary to perform these procedures at unscheduled time points if deemed clinically necessary by the investigator.

Furthermore, additional evaluations/testing may be deemed necessary by the investigator and or the Sponsor for reasons related to subject safety. In some cases, such evaluation/testing may be potentially sensitive in nature (e.g., HIV, Hepatitis C, etc.), and thus local regulations may require that additional informed consent be obtained from the subject. In these cases, such evaluations/testing will be performed in accordance with those regulations.

7.1.1 Administrative Procedures

7.1.1.1 Informed Consent

The investigator or qualified designee must obtain documented consent from each potential subject or each subject's legally acceptable representative prior to participating in a clinical trial or Future Biomedical Research. If there are changes to the subject's status during the trial (e.g., health or age of majority requirements), the investigator or qualified designee must ensure the appropriate consent is in place.

7.1.1.1.1 General Informed Consent

Consent must be documented by the subject's dated signature or by the subject's legally acceptable representative's dated signature on a consent form along with the dated signature of the person conducting the consent discussion.

A copy of the signed and dated consent form should be given to the subject before participation in the trial.

The initial informed consent form, any subsequent revised written informed consent form and any written information provided to the subject must receive the IRB/ERC's approval/favorable opinion in advance of use. The subject or his/her legally acceptable representative should be informed in a timely manner if new information becomes available that may be relevant to the subject's willingness to continue participation in the trial. The communication of this information will be provided and documented via a revised consent form or addendum to the original consent form that captures the subject's dated signature or by the subject's legally acceptable representative's dated signature.

Specifics about a trial and the trial population will be added to the consent form template at the protocol level.

The informed consent will adhere to IRB/ERC requirements, applicable laws and regulations and Sponsor requirements.

7.1.1.1.2 Consent and Collection of Specimens for Future Biomedical Research

The investigator or qualified designee will explain the Future Biomedical Research consent to the subject, answer all of his/her questions, and obtain written informed consent before performing any procedure related to the Future Biomedical Research sub-trial. A copy of the informed consent will be given to the subject.

7.1.1.2 Inclusion/Exclusion Criteria

All inclusion and exclusion criteria will be reviewed at Visit 1 and 2 by the investigator or qualified designee to ensure that the subject qualifies for the trial.

7.1.1.3 Subject Identification Card

All subjects will be given a Subject Identification Card identifying them as participants in a research trial. The card will contain trial site contact information (including direct telephone numbers) to be utilized in the event of an emergency. The investigator or qualified designee will provide the subject with a Subject Identification Card immediately after the subject provides written informed consent. At the time of treatment allocation/randomization, site personnel will add the treatment/randomization number to the Subject Identification Card.

The subject identification card also contains contact information for the emergency unblinding call center so that a health care provider can obtain information about trial medication/vaccination in emergency situations where the investigator is not available.

7.1.1.4 Medical History

A medical history will be obtained by the investigator or qualified designee.

The medical indication for surgery and corresponding surgical procedure will be entered into the database.

7.1.1.5 Prior and Concomitant Medications Review

7.1.1.5.1 Prior Medications

The investigator or qualified designee will review prior medication use, including any protocol-specified washout requirement, and record prior medication taken by the subject within 14 days before starting the trial. See Section 5.5 for concomitant medication information.

7.1.1.5.2 Concomitant Medications

The investigator or qualified designee will record any medication (e.g., beta-blockers, calcium channel blockers, anti-arrhythmic agents) taken by or administered to the subject during surgery and up to 14 days after surgery. See Section 5.5 for concomitant medication information. Rocuronium or vecuronium, are concomitant medications to be used per label as adjunct to general anesthesia.

7.1.1.6 Assignment of Screening Number

All consented subjects will be given a unique screening number that will be used to identify the subject for all procedures that occur prior to randomization or treatment allocation. Each subject will be assigned only one screening number. Screening numbers must not be re-used for different subjects.

Any subject who is screened multiple times will retain the original screening number assigned at the initial screening visit.

Specific details on the screening visit requirements (screening/rescreening) are provided in Section 7.1.5.1.

7.1.1.7 Assignment of Treatment/Randomization Number

All eligible subjects will be randomly allocated and will receive a treatment/randomization number. The treatment/randomization number identifies the subject for all procedures occurring after treatment allocation/randomization. Once a treatment/randomization number is assigned to a subject, it can never be re-assigned to another subject.

A single subject cannot be assigned more than 1 treatment/randomization number.

7.1.1.8 Trial Compliance (Medication)

Compliance with dosage will be based on the actual dosage of study medication administered versus assigned dosage. For further information, refer to Section 8.11.

7.1.2 Clinical Procedures/Assessments

7.1.2.1 Physical Examination

The principal investigator or licensed clinician (i.e., physician, physician's assistant, or nurse practitioner) will perform a general physical examination of the following organ systems at Visit 1:

- Head, Eyes, Ears, Nose, and Throat
- Neck
- Respiratory system
- Cardiovascular system
- Abdomen
- Skin and extremities
- Neurological system, including mental status, motor strength, muscle tone, sensation, and reflexes.

Any medical conditions found during the screening physical exam will be recorded in the Sponsor database.

A second physical examination will be performed at the post-anesthetic visit by the BSA. Any clinically significant change from the first physical examination (screening) will be recorded in the Sponsor database.

7.1.2.2 Body Weight and Height

Body weight and height will be collected and recorded in the Sponsor database. Measurements should be rounded to the nearest whole number. Body weight will be obtained without shoes and with heavy clothing (e.g., jacket or coat) removed.

7.1.2.3 Body Mass Index

Body mass index will be calculated at Visit 1 and Visit 2 to ensure subjects meet eligibility criteria. The formula for BMI is weight in kilograms divided by height in meters squared (kg/m^2).

7.1.2.4 Electrocardiogram

Visit 1:

A 12-lead ECG will be performed at Visit 1 for documentation of baseline rhythm and cardiac disease. Historical ECGs (those done prior to informed consent) are not acceptable.

To assist sites with scheduling study procedures, the screening ECG may be performed at Visit 2 if necessary. However, it must be performed prior to induction of anesthesia or prior to administration of study medication.

Visit 2:

During the peri-anesthetic visit, continuous ECG monitoring will occur at least 5 minutes before, during, and for 30 minutes after administration of study medication (sugammadex or neostigmine) to identify events of treatment emergent sinus bradycardia, treatment emergent sinus tachycardia, or other treatment emergent cardiac arrhythmias, which will be recorded in the Sponsor database. Sites should also record the heart rate and associated blood pressure for these events.

Events of clinically relevant sinus bradycardia, clinically relevant sinus tachycardia, or other clinically relevant cardiac arrhythmias, (according to the definitions in Section 4.2.3.2), should be reported as ECIs in the Sponsor database.

Printing of the ECG trace is not required per protocol.

7.1.2.5 Vital Signs

Heart rate, blood pressure, respiratory rate, oxygen saturation, and temperature will be obtained at the following time points as shown in Table 2.

Table 2 Vital Sign Measurements

**Vital Sign Measurement**	**Visit 1**	**Visit 2**			**Visit 3**
		Prior to administration of NMBA	After the last dose of NMBA is administered and (approximately 5 minutes) prior to administration of study medication	At 2, 5, 10, 30 minutes following administration of study medication	
**HR**	X	X	X	X	X
**BP**	X	X	X	X	X
**RR**	X	X	X	X	X
**O_2 saturation**	X	X	X	X	X
**Body Temperature:** Obtained per local guidelines/standard methods, except when noted*.	X	X	X<br>***Required: core body temperature**[a, b]	X	X

[a] The target core body temperature is ≥35ºC (95ºF).

[b] The only acceptable locations for core body temperature measurement are: nasopharynx, esophagus, bladder, pulmonary artery branch, or rectum.

Abbreviations: BP= blood pressure, HR= heart rate, NMBA = neuromuscular blocking agent, 0_2= oxygen, RR= respiratory rate

7.1.2.6 Administration of Neuromuscular Blocking Agents

NMBAs will be dosed as indicated per prescribing information for intubation purposes and maintenance of neuromuscular block by re-dosing or continuous infusion.

Only one NMBA should be used for the entire duration of surgery, including the intubation dose. The NMBA used during surgery should be the NMBA noted in the IVRS confirmation.

7.1.2.7 Neuromuscular Monitoring

Neuromuscular monitoring will be performed using locally available neuromuscular transmission monitoring (NMTM, e.g., objective NMTM using a quantitative TOF-Watch or subjective NMTM using a peripheral nerve stimulator).

For subjects randomized to moderate block, after the last dose of NMBA and within 5 minutes of reappearance of T2, sugammadex or neostigmine will be given, according to the randomization schedule.

For subjects randomized to deep block, after the last dose of NMBA and within 5 minutes of detection of PTC $\geq$1, sugammadex will be given.

Clinical evidence of recovery will be assessed using the standard practice for monitoring in effect at the site.

7.1.3 Laboratory Procedures/Assessments

Details regarding specific laboratory procedures/assessments to be performed in this trial are provided below. The total amount of blood to be drawn/collected over the course of the trial (from pre-trial to post-trial visits), including approximate blood volumes drawn/collected by visit and by sample type per subject can be found in Section 12.3.

7.1.3.1 Laboratory Safety Evaluations (Hematology and Chemistry)

Laboratory tests for hematology and chemistry are specified in Table 3.

Table 3 Laboratory Tests

Hematology	Chemistry	Other
Hematocrit	Albumin	Urine pregnancy test
Hemoglobin	Alkaline phosphatase	Serum β-human chorionic gonadotropin (β-hCG)
Platelet count	Alanine aminotransferase	
WBC (total and differential)	Aspartate aminotransferase	
	Bicarbonate	
	Calcium	
	Chloride	
	Creatinine	
	Glucose	
	Phosphorus	
	Potassium	
	Sodium	
	Total Bilirubin	
	Direct Bilirubin, if total bilirubin is elevated above the upper limit of normal	
	Total protein	
	Blood Urea Nitrogen	

All laboratory samples will be collected as per Section 6.0 – Trial Flow Chart. Safety laboratory samples will be sent to a central laboratory for analysis. Safety labs should be obtained pre-administration of NMBA and post-operative safety labs should be obtained within 4-6 hours after administration of study medication. Detailed instructions for specimen collection, packaging, and shipment will be provided in a Laboratory Manual by the Central Laboratory.

7.1.3.2 Pregnancy Tests

Female subjects of child-bearing potential are required to have a negative pregnancy test within 24 hours prior to surgery. At Visit 1, sites may test female subjects using a urine pregnancy test (provided by central lab) or a serum β-human chorionic gonadotropin pregnancy test if part of their standard of care process (results analyzed at a local lab).

If Visit 1 occurs more than 24 hours prior to surgery, the pregnancy test will need to be repeated within 24 hours prior to surgery.

If a urine pregnancy test is positive, then a serum β-hCG pregnancy test is required unless local requirements require otherwise. This test may be performed locally. Results (negative for pregnancy) must be received by site staff before the subject is given study medication.

If serum pregnancy is positive, the subject is to be discontinued from the trial.

7.1.3.3 Future Biomedical Research Samples

The following specimens are to be obtained as part of Future Biomedical Research:

- DNA for future research

7.1.4 Other Procedures

7.1.4.1 Withdrawal/Discontinuation

Once a subject receives study medication at Visit 2, all applicable procedures at subsequent visits should be performed as per study flow chart.

Any adverse events which are present at the time of withdrawal should be followed in accordance with the safety requirements outlined in Section 7.2 - Assessing and Recording Adverse Events.

7.1.4.1.1 Withdrawal From Future Biomedical Research

Subjects may withdraw their consent for Future Biomedical Research. Subjects may withdraw consent at any time by contacting the principal investigator for the main trial. If medical records for the main trial are still available, the investigator will contact the Sponsor using the designated mailbox (clinical.specimen.management@merck.com). Subsequently, the subject's consent for Future Biomedical Research will be withdrawn. A letter will be sent from the Sponsor to the investigator confirming the withdrawal. It is the responsibility of the investigator to inform the subject of completion of withdrawal. Any analyses in progress at the time of request for withdrawal or already performed prior to the request being received by the Sponsor will continue to be used as part of the overall research trial data and results. No new analyses would be generated after the request is received.

In the event that the medical records for the main trial are no longer available (e.g., if the investigator is no longer required by regulatory authorities to retain the main trial records) or the specimens have been completely anonymized, there will no longer be a link between the subject's personal information and their specimens. In this situation, the request for specimen withdrawal cannot be processed.

7.1.4.1.2 Lost to Follow-up

If a subject fails to complete a required study visit (e.g., follow-up safety contact) and/or if the site is unable to contact the subject, the following procedures are to be performed:

- The site must attempt to contact the subject and reschedule the missed visit.
- The investigator or designee must make every effort to regain contact with the subject (e.g., phone calls and/or a certified letter to the subject's last known mailing address or locally equivalent methods). These contact attempts should be documented in the subject's medical record.

Note: A subject is not considered lost to follow-up until the last scheduled visit for the individual subject. The amount of missing data for the subject will be managed via the pre-specified data handling and analysis guidelines.

7.1.4.1.3 Subject Blinding/Unblinding

When the investigator or delegate needs to identify the drug used by a subject and the dosage administered in case of emergency e.g., the occurrence of serious adverse experiences, he/she will contact the emergency unblinding call center by telephone and make a request for emergency unblinding. As requested by the investigator or delegate the emergency unblinding call center will provide the information to him/her promptly and report unblinding to the sponsor. Prior to contacting the emergency unblinding call center to request unblinding of a subject's treatment assignment, the investigator or delegate must enter the intensity of the adverse experiences observed, the relation to study drug, the reason thereof, etc., in the medical chart etc. Subjects whose treatment assignment has been unblinded by the investigator/delegate and/or non-study treating physician must be discontinued from study drug, but should continue to be monitored in the trial.

Additionally, the investigator must go into the IVRS system and perform the unblind in the IVRS system to update drug disposition. In the event that the emergency unblinding call center is not available for a given site in this trial, IVRS/IWRS should be used for emergency unblinding in the event that this is required for subject safety.

Treatment/Vaccine identification information is to be unmasked ONLY if necessary for the welfare of the subject. Every effort should be made not to unblind the subject.

In the event that unblinding has occurred, the circumstances around the unblinding (e.g., date, reason and person performing the unblinding) must be documented promptly, and the Sponsor Clinical Director notified as soon as possible. Only the principal investigator or delegate and the respective subject's code should be unblinded. Other trial site personnel and Sponsor personnel directly associated with the conduct of the trial should not be unblinded. Subjects whose treatment assignment has been unblinded by the investigator/delegate and/or non-study treating physician must be discontinued from study drug, but should continue to be monitored in the trial.

7.1.4.2 Calibration of Equipment

The investigator or qualified designee has the responsibility to ensure that any device or instrument used for a clinical evaluation/test during a clinical study that provides information about inclusion/exclusion criteria and/or safety or efficacy parameters shall be suitably calibrated and/or maintained to ensure that the data obtained is reliable and/or reproducible. Documentation of equipment calibration must be retained as source documentation at the study site.

7.1.5 Visit Requirements

Visit requirements are outlined in Section 6.0 - Trial Flow Chart. Specific procedure-related details are provided above in Section 7.1 - Trial Procedures.

7.1.5.1 Screening Visit (V1)

Potential subjects will be evaluated to determine if they fulfill the entry requirements as set forth in Section 5.1. Screening procedures may be repeated after consultation with the Sponsor.

A subject may be rescreened, but the Sponsor should be consulted prior to any rescreen. Rescreens will be handled on a case by case basis. Only one rescreen is allowed per subject.

7.1.5.2 Peri-anesthetic Visit (V2)

The following procedures will be performed during the Peri-anesthetic visit:

1. Confirm the subject still meets inclusion/exclusion criteria.
2. Measure subject weight
3. Randomize the subject using IVRS. Trial treatment should begin within 1 day of randomization.

   a) Randomization 1 day before surgery (Visit 2 performed across 2 days)

   In certain situations (e.g., if surgery is scheduled for early morning), randomization in IVRS may occur 1 day in advance of the scheduled surgery only if the following procedures are completed prior to calling into IRT for randomization:

   1. Confirm the subject meets inclusion/exclusion criteria (including recalculation of BMI using new weight measurement)
   2. Take subject weight and recalculate BMI

   If procedures above cannot be completed prior to randomizing the subject in IVRS, then randomization in IVRS cannot occur 1 day in advance of the surgery.

   *Visit 1 (V1) and Visit 2 (V2) should be conducted on separate days however in certain situations this may not be possible. For these exceptions, V1 and V2 may be conducted on the same day. Refer to guidance below.

   b) *V1 and V2: both visits performed in full on the same day

   If V1 and V2 are conducted on the same day (including surgery), the following procedures need only be done once:

   1. Inclusion/exclusion criteria review
   2. Concomitant medication review
   3. Weight measurement and BMI calculation

   All other protocol procedures (e.g., vital signs, safety labs) must be conducted as indicated in the trial flow chart (Section 6.0) and as outlined in Section 7.1.5.2.

c) ***V1 and V2: part of Visit 2 performed same day as V1 but surgery is scheduled for the next day (i.e., Visit 2 performed across 2 days)**

If V1 and part of V2 are conducted on the same day (not including surgery), the following procedures should be conducted prior to calling into IRT and only need to be done once.

1. Inclusion/exclusion criteria review
2. Concomitant medication review
3. Weight measurement and BMI calculation

All other protocol procedures (e.g., vital signs, safety labs) must be conducted as indicated in the trial flow chart (Section 6.0) and as outlined as outlined in Section 7.1.5.2.

d) **If surgery is postponed more than one day after randomization in IVRS**:

- Sites must contact the Sponsor if a subject‘s surgery is postponed for more than one day from the date the subject is randomized in IVRS (e.g., surgery postponed due to subject illness).
- Trial status of subjects in these situations will be handled on a case by case basis in consultation with the Sponsor.

4. Pharmacist (or delegate) prepares study medication (refer to Study Drug Manual for details)
5. Insert an IV cannula for the administration of anesthetic drugs, NMBA, and study medication.

<u>Administration of anesthesia:</u>

6. Anesthesia will be induced and maintained according to the need of the subject.

<u>Before administration of the NMBA:</u>

7. Vital signs (per protocol) will be recorded.
8. A baseline blood sample for safety analysis will be obtained and sent to the central lab vendor.
9. A blood sample will be collected for future biomedical research (if consent obtained).
10. Neuromuscular monitoring will be performed using locally available NMTM (e.g., objective NMTM or subjective NMTM using a peripheral nerve stimulator [PNS]).

<u>Administration of NMBA until administration of study medication:</u>

11. Administer NMBA for intubation. Only one NMBA should be used for the entire duration of surgery. The NMBA used during surgery should be the NMBA noted in the IVRS.
12. Additional doses of NMBA will be administered as clinically required for the duration of the surgery to target maintenance of the assigned depth of block, as determined by the Primary Investigator or designee.

13. Vital signs (per protocol) will be recorded.
14. Record a core body temperature after the last dose of NMBA is administered and at the time depth of block is assessed prior to administration of sugammadex or neostigmine.

<u>From administration of study medication (sugammadex or neostigmine) until the end of anesthesia</u>:

15. For subjects randomized to moderate block: after last dose of administered NMBA and within 5 minutes of reappearance of T2 (with a lower limit of TOF count 1 and upper limit of TOF count of 4), study medication (sugammadex or neostigmine) will be administered.
16. For subjects randomized to deep block: after last dose of administered NMBA and within 5 minutes of detection of PTC ≥1 and TOF Count of 0, study medication (sugammadex) will be administered.
17. Study staff should NOT record 'sugammadex' or 'neostigmine' for annotation of study medication administration. To maintain blinding in the anesthetic chart, only indicate 'study medication' or 'SM' in the place of sugammadex or neostigmine.
18. Continuous ECG monitoring: at least 5 minutes before, during, and for 30 minutes after administration of study medication.
19. The following additional study procedures should be done on the following schedule:

Time After Study Medication	2 mins	5 mins	10 mins	30 mins	4-6 hrs
Vital Signs	X	X	X	X	
Blood Samples for Safety Labs					X

20. Continue neuromuscular monitoring using locally available NMTM (e.g., objective NMTM using a quantitative TOF watch or subjective NMTM using a peripheral nerve stimulator) until the subject is ready to be extubated.
21. Adverse event(s) and concomitant medication(s) will be recorded.

7.1.5.3 Post-Anesthetic Visit (V3)

Visit 3 should be conducted at least 4 hours following study medication administration up to a maximum of 36 hours after study medication administration (or before discharge, whichever is earlier) (see Trial Flowchart – Section 6.0).

The BSA will review the subject's medical records in order to properly assess the subject at this visit with the following exceptions: study medication assignment, depth of NMB, and drug preparation records.

7.1.5.4 Follow-up Safety Contact (V4)

Approximately 14 days post study medication administration, subjects will be contacted by designated site personnel by telephone (or in person if the subject is still in the hospital). New and ongoing AEs and concomitant medication(s) should be assessed and reported in the database.

7.2 Assessing and Recording Adverse Events and Patient/Device Events

An adverse event is defined as any untoward medical occurrence in a patient or clinical investigation subject administered a pharmaceutical product and which does not necessarily have to have a causal relationship with this treatment. An adverse event can therefore be any unfavourable and unintended sign (including an abnormal laboratory finding, for example), symptom, or disease temporally associated with the use of a medicinal product or protocol-specified procedure, whether or not considered related to the medicinal product or protocol-specified procedure. Any worsening (i.e., any clinically significant adverse change in frequency and/or intensity) of a preexisting condition that is temporally associated with the use of the Sponsor's product, is also an adverse event.

Changes resulting from normal growth and development that do not vary significantly in frequency or severity from expected levels are not to be considered adverse events. Examples of this may include, but are not limited to, teething, typical crying in infants and children and onset of menses or menopause occurring at a physiologically appropriate time.

Sponsor's product includes any pharmaceutical product, biological product, device, diagnostic agent or protocol-specified procedure, whether investigational (including placebo or active comparator medication) or marketed, manufactured by, licensed by, provided by or distributed by the Sponsor for human use.

Adverse events may occur during clinical trials, or as prescribed in clinical practice, from overdose (whether accidental or intentional), from abuse and from withdrawal.

All adverse events that occur after the consent form is signed but before treatment allocation/randomization must be reported by the investigator if they cause the subject to be excluded from the trial, or are the result of a protocol-specified intervention, including but not limited to washout or discontinuation of usual therapy, diet, placebo treatment or a procedure. From the time of treatment allocation/randomization through 14 days following cessation of treatment, all adverse events must be reported by the investigator. Such events will be recorded at each examination on the Adverse Event case report forms/worksheets. The reporting timeframe for adverse events meeting any serious criteria is described in section 7.2.3.1. The investigator will make every attempt to follow all subjects with non-serious adverse events for outcome.

Electronic reporting procedures can be found in the Electronic Data Capture (EDC) data entry guidelines. Paper reporting procedures can be found in the Investigator Trial File Binder (or equivalent).

Device and/or patient events include all untoward events related to the use of the device or device-like features of a drug delivery system. Device events include any malfunction or deterioration in the characteristics and/or performance of the device, as well as any

inadequacy in the labeling or the instructions for use, that led to or could have led to an untoward event for the user or any person. Patient events are adverse events experienced by the subject caused by or suspected to be caused by the device or device-like features of a drug delivery system.

From the time of allocation/randomization through 14 days following cessation of treatment, all device or patient events must be reported by the investigator. Such events will be recorded at each examination on the Adverse Event case report forms/worksheets. The reporting timeframe for events meeting any serious criteria is described in section 7.2.3.1. The investigator will make every attempt to follow all non-serious device or subject events for outcome.

Electronic reporting procedures can be found in the Electronic Data Capture (EDC) data entry guidelines. Paper reporting procedures can be found in the Investigator Trial File Binder (or equivalent).

During the course of the trial, a subject may provide feedback related to the device or device-like features of a drug delivery system. This "customer feedback" is defined as a report that does not allege a product quality complaint or defect and has no relevant safety information/untoward event associated with it (e.g., goodwill or courtesy replacement, consumer preference or suggestion, remark which may suggest an improvement in the functionality or quality of a medical device or device-like features of a drug delivery system). All reports of customer feedback must be reported to the Sponsor. Sponsor Contact information can be found in the Investigator Trial File Binder (or equivalent).

7.2.1 Definition of an Overdose for This Protocol and Reporting of Overdose to the Sponsor

In this trial, an overdose of sugammadex is considered a dose ≥ 2 X the intended dose to which the subject was randomized. In this trial, an overdose of neostigmine is any dose greater than 5.0 mg as indicated in the labeling of neostigmine. An overdose of glycopyrrolate is considered a dose of greater than 2.0 mg.

If an adverse event(s) is associated with ("results from") the overdose of Sponsor's product or vaccine, the adverse event(s) is reported as a non-serious adverse event, unless other serious criteria are met.

If a dose of Sponsor's product or vaccine meeting the protocol definition of overdose is taken without any associated clinical symptoms or abnormal laboratory results, the overdose is reported as a non-serious adverse events using the terminology "accidental or intentional overdose without adverse effect."

All reports of overdose with and without an adverse event must be reported by the investigator within 24 hours to the Sponsor either by electronic media or paper. Electronic reporting procedures can be found in the EDC data entry guidelines. Paper reporting procedures can be found in the Investigator Trial File Binder (or equivalent).

7.2.2 Reporting of Pregnancy and Lactation to the Sponsor

Although pregnancy and lactation are not considered adverse events, it is the responsibility of investigators or their designees to report any pregnancy or lactation in a subject (spontaneously reported to them) that occurs during the trial.

Pregnancies and lactations that occur after the consent form is signed but before treatment allocation/randomization must be reported by the investigator if they cause the subject to be excluded from the trial, or are the result of a protocol-specified intervention, including but not limited to washout or discontinuation of usual therapy, diet, placebo treatment or a procedure. Pregnancies and lactations that occur from the time of treatment allocation/randomization through 14 days following cessation of Sponsor's product must be reported by the investigator. All reported pregnancies must be followed to the completion/termination of the pregnancy. Pregnancy outcomes of spontaneous abortion, missed abortion, benign hydatidiform mole, blighted ovum, fetal death, intrauterine death, miscarriage and stillbirth must be reported as serious events (Important Medical Events). If the pregnancy continues to term, the outcome (health of infant) must also be reported.

Such events must be reported within 24 hours to the Sponsor either by electronic media or paper. Electronic reporting procedures can be found in the EDC data entry guidelines. Paper reporting procedures can be found in the Investigator Trial File Binder (or equivalent).

7.2.3 Immediate Reporting of Adverse Events and Incidents to the Sponsor

7.2.3.1 Serious Adverse Events and Incidents

A serious adverse event is any adverse event occurring at any dose or during any use of Sponsor's product that:

- Results in death;
- Is life threatening;
- Results in persistent or significant disability/incapacity;
- Results in or prolongs an existing inpatient hospitalization;
- Is a congenital anomaly/birth defect;
- Is an other important medical event.

Note: In addition to the above criteria, adverse events meeting either of the below criteria, although not serious per ICH definition, are reportable to the Sponsor in the same timeframe as SAEs to meet certain local requirements.

- Is a cancer;
- Is associated with an overdose.

Refer to Table 4 for additional details regarding each of the above criteria.

For the time period beginning when the consent form is signed until treatment allocation/randomization, any serious adverse event, or follow up to a serious adverse event, including death due to any cause, that occurs to any subject must be reported within 24 hours

to the Sponsor if it causes the subject to be excluded from the trial, or is the result of a protocol-specified intervention, including but not limited to washout or discontinuation of usual therapy, diet, placebo treatment or a procedure.

For the time period beginning at treatment allocation/randomization through 14 days following cessation of treatment, any serious adverse event, or follow up to a serious adverse event, including death due to any cause, whether or not related to the Sponsor's product, must be reported within 24 hours to the Sponsor either by electronic media or paper. Electronic reporting procedures can be found in the EDC data entry guidelines. Paper reporting procedures can be found in the Investigator Trial File Binder (or equivalent).

Additionally, any serious adverse event, considered by an investigator who is a qualified physician to be related to the Sponsor's product that is brought to the attention of the investigator at any time outside of the time period specified in the previous paragraph also must be reported immediately to the Sponsor.

All subjects with serious adverse events must be followed up for outcome.

An incident is any malfunction or deterioration in the characteristics and/or performance of the device, as well as any inadequacy in the labeling or the instructions for use that, directly or indirectly, led to or could have led to, the death of a subject or user, or of other persons, or to a serious deterioration in their state of health.

A serious deterioration in the state of health can include:

- Life-threatening illness, even if temporary in nature;
- Permanent impairment of a body function or permanent damage to a body structure;
- Any indirect harm as a consequence of an incorrect diagnostic or IVD test results when used within instructions for use;
- Fetal distress, fetal death or any congenital abnormalities or birth defects;
- Condition necessitating medical or surgical intervention, including hospitalization or prolongation or existing hospitalization to prevent one of the above;
- Cases that are considered medically significant

For the time period beginning at treatment allocation/randomization through 14 days following cessation of treatment, any incident, or follow-up to an incident, whether or not related to the device, must be reported within 24 hours to the Sponsor either by electronic media or paper. Electronic reporting procedures can be found in the EDC data entry guidelines. Paper reporting procedures can be found in the Investigator Trial File Binder (or equivalent).

Additionally, any incident considered by an investigator who is a qualified physician to be related to the Sponsor's product that is brought to the attention of the investigator at any time outside of the time period specified above also must be reported immediately to the Sponsor.

All subjects involved with incidents must be followed up for outcome.

7.2.3.2 Events of Clinical Interest

Selected non-serious and serious adverse events are also known as Events of Clinical Interest (ECI) and must be reported to the Sponsor.

For the time period beginning when the consent form is signed until treatment allocation/randomization, any ECI, or follow up to an ECI, that occurs to any subject must be reported within 24 hours to the Sponsor if it causes the subject to be excluded from the trial, or is the result of a protocol-specified intervention, including but not limited to washout or discontinuation of usual therapy, diet, placebo treatment or a procedure.

For the time period beginning at treatment allocation/randomization through 14 days following cessation of treatment, any ECI, or follow up to an ECI, whether or not related to the Sponsor's product, must be reported within 24 hours to the Sponsor, either by electronic media or paper. Electronic reporting procedures can be found in the EDC data entry guidelines. Paper reporting procedures can be found in the Investigator Trial File Binder (or equivalent).

Events of clinical interest for this trial include:

1. an elevated AST or ALT lab value that is greater than or equal to 3X the upper limit of normal and an elevated total bilirubin lab value that is greater than or equal to 2X the upper limit of normal and, at the same time, an alkaline phosphatase lab value that is less than 2X the upper limit of normal, as determined by way of protocol-specified laboratory testing or unscheduled laboratory testing.*

   *Note: These criteria are based upon available regulatory guidance documents. The purpose of the criteria is to specify a threshold of abnormal hepatic tests that may require an additional evaluation for an underlying etiology. The trial site guidance for assessment and follow up of these criteria can be found in the Investigator Trial File Binder (or equivalent).

2. Clinically relevant arrhythmias, inclusive of clinically relevant sinus bradycardia and clinically relevant sinus tachycardia

   - **Clinically relevant sinus bradycardia** is defined as any bradycardia event necessitating intervention, as determined by investigator judgment.
   - **Clinically relevant sinus tachycardia** is defined as any tachycardia event necessitating intervention, as determined by investigator judgment.
   - **Other clinically relevant cardiac arrhythmia** is defined as any arrhythmia event necessitating intervention, as determined by investigator judgment (i.e., new or worsening atrial fibrillation, atrial tachycardia, ventricular tachycardia, or ventricular fibrillation, etc).

3. Hypersensitivity and/or anaphylaxis

- **Hypersensitivity**: The term hypersensitivity describes objectively reproducible symptoms and signs of allergic disease initiated by exposure to a defined stimulus at a dose tolerated by normal persons. Refer to the ECI Guidance document (or equivalent) for more information.
- **Anaphylaxis**: The term anaphylaxis is an umbrella term for a serious, life-threatening generalized or systemic hypersensitivity reaction that is rapid in onset. For the purpose of this study, adjudication of potential cases of anaphylaxis is defined by Sampson et al. [4].

7.2.4 Evaluating Adverse Events

An investigator who is a qualified physician will evaluate all adverse events with respect to the elements outlined in Table 4. The investigator's assessment of causality is required for each adverse event. Refer to Table 4 for instructions in evaluating adverse events.

Table 4 Evaluating Adverse Events

**Maximum Intensity**	**Mild**	awareness of sign or symptom, but easily tolerated (for pediatric trials, awareness of symptom, but easily tolerated)
	**Moderate**	discomfort enough to cause interference with usual activity (for pediatric trials, definitely acting like something is wrong)
	**Severe**	incapacitating with inability to work or do usual activity (for pediatric trials, extremely distressed or unable to do usual activities)
**Seriousness**	A serious adverse event (AE) is any adverse event occurring at any dose or during any use of Sponsor's product that:	
	**†Results in death**; or	
	**†Is life threatening; or** places the subject, in the view of the investigator, at immediate risk of death from the event as it occurred [Note: This does not include an adverse event that, had it occurred in a more severe form, might have caused death.]; or	
	**†Results in a persistent or significant disability/incapacity** (substantial disruption of one's ability to conduct normal life functions); or	
	**†Results in or prolongs an existing inpatient hospitalization** (hospitalization is defined as an inpatient admission, regardless of length of stay, even if the hospitalization is a precautionary measure for continued observation. (Note: Hospitalization for an elective procedure to treat a pre-existing condition that has not worsened is not a serious adverse event. A pre-existing condition is a clinical condition that is diagnosed prior to the use of a Merck product and is documented in the patient's medical history.); or	
	**†Is a congenital anomaly/birth defect (**in offspring of subject taking the product regardless of time to diagnosis); or	
	**Is a cancer** (although not serious per ICH definition, is reportable to the Sponsor within 24 hours to meet certain local requirements); **or**	
	Overdose, although not serious per ICH definition, whether accidental or intentional, with or without an accompanying adverse event/serious adverse event, is reportable to the Sponsor within 24 hours to meet certain local requirements.	
	**Other important medical events** that may not result in death, not be life threatening, or not require hospitalization may be considered a serious adverse event when, based upon appropriate medical judgment, the event may jeopardize the subject and may require medical or surgical intervention to prevent one of the outcomes listed previously (designated above by a †).	
**Duration**	Record the start and stop dates of the adverse event. If less than 1 day, indicate the appropriate length of time and units	
**Action taken**	Did the adverse event cause the Sponsor's product to be discontinued?	
**Relationship to Sponsor's Product**	Did the Sponsor's product cause the adverse event? The determination of the likelihood that the Sponsor's product caused the adverse event will be provided by an investigator who is a qualified physician. The investigator's signed/dated initials on the source document or worksheet that supports the causality noted on the AE form, ensures that a medically qualified assessment of causality was done. This initialed document must be retained for the required regulatory time frame. The criteria below are intended as reference guidelines to assist the investigator in assessing the likelihood of a relationship between the test drug and the adverse event based upon the available information<br>**The following components are to be used to assess the relationship between the Sponsor's product and the AE**; the greater the correlation with the components and their respective elements (in number and/or intensity), the more likely the Sponsor's product caused the adverse event:	
	**Exposure**	Is there evidence that the subject was actually exposed to the Sponsor's product such as: reliable history, acceptable compliance assessment (pill count, diary, etc.), expected pharmacologic effect, or measurement of drug/metabolite in bodily specimen?
	**Time Course**	Did the AE follow in a reasonable temporal sequence from administration of the Sponsor's product? Is the time of onset of the AE compatible with a drug-induced effect (applies to trials with investigational medicinal product)?
	**Likely Cause**	Is the AE not reasonably explained by another etiology such as underlying disease, other drug(s)/vaccine(s), or other host or environmental factors

<table>
<tr><td rowspan="3">Relationship to Sponsor's Product (continued)</td><td colspan="2">The following components are to be used to assess the relationship between the Sponsor's product and the AE: (continued)</td></tr>
<tr><td>Dechallenge</td><td>Was the Sponsor's product discontinued or dose/exposure/frequency reduced?
If yes, did the AE resolve or improve?
If yes, this is a positive dechallenge. If no, this is a negative dechallenge.
(Note: This criterion is not applicable if: (1) the AE resulted in death or permanent disability; (2) the AE resolved/improved despite continuation of the Sponsor's product; (3) the trial is a single-dose drug trial); or (4) Sponsor's product(s) is/are only used one time.)</td></tr>
<tr><td>Rechallenge</td><td>Was the subject re-exposed to the Sponsor's product in this trial?
If yes, did the AE recur or worsen?
If yes, this is a positive rechallenge. If no, this is a negative rechallenge.
(Note: This criterion is not applicable if: (1) the initial AE resulted in death or permanent disability, or (2) the trial is a single-dose drug trial); or (3) Sponsor's product(s) is/are used only one time.)
NOTE: IF A RECHALLENGE IS PLANNED FOR AN ADVERSE EVENT WHICH WAS SERIOUS AND WHICH MAY HAVE BEEN CAUSED BY THE SPONSOR'S PRODUCT, OR IF RE-EXPOSURE TO THE SPONSOR'S PRODUCT POSES ADDITIONAL POTENTIAL SIGNIFICANT RISK TO THE SUBJECT THEN THE RECHALLENGE MUST BE APPROVED IN ADVANCE BY THE SPONSOR CLINICAL DIRECTOR AND THE INSTITUTIONAL REVIEW BOARD/INDEPENDENT ETHICS COMMITTEE.</td></tr>
<tr><td></td><td>Consistency with Trial Treatment Profile</td><td>Is the clinical/pathological presentation of the AE consistent with previous knowledge regarding the Sponsor's product or drug class pharmacology or toxicology?</td></tr>
<tr><td colspan="3">The assessment of relationship will be reported on the case report forms /worksheets by an investigator who is a qualified physician according to his/her best clinical judgment, including consideration of the above elements.</td></tr>
<tr><td colspan="2">Record one of the following:</td><td>Use the following scale of criteria as guidance (not all criteria must be present to be indicative of a Sponsor's product relationship).</td></tr>
<tr><td colspan="2">Yes, there is a reasonable possibility of Sponsor's product relationship.</td><td>There is evidence of exposure to the Sponsor's product. The temporal sequence of the AE onset relative to the administration of the Sponsor's product is reasonable. The AE is more likely explained by the Sponsor's product than by another cause.</td></tr>
<tr><td colspan="2">No, there is not a reasonable possibility of Sponsor's product relationship</td><td>Subject did not receive the Sponsor's product OR temporal sequence of the AE onset relative to administration of the Sponsor's product is not reasonable OR the AE is more likely explained by another cause than the Sponsor’s product. (Also entered for a subject with overdose without an associated AE.)</td></tr>
</table>

7.2.5 Sponsor Responsibility for Reporting Adverse Events

All Adverse Events will be reported to regulatory authorities, IRB/IECs and investigators in accordance with all applicable global laws and regulations, i.e., per ICH Topic E6 (R1) Guidelines for Good Clinical Practice.

7.3 TRIAL GOVERNANCE AND OVERSIGHT

7.3.1 Clinical Adjudication Committee

A Clinical Adjudication Committee (CAC) will evaluate the following events for the purposes of confirming them according to the criteria in Section 8.0 – Statistical Analysis Plan, as well as evaluating the presence of confounding factors.

This external committee of independent consultants will assess the following events:

- Hypersensitivity and/or anaphylaxis

The BSA and/or Principal Investigator (or delegate) will identify events to be adjudicated. In addition, the Sponsor will periodically review the blinded clinical database in order to identify events that may meet criteria for adjudication. Upon identification of such an event, the Sponsor may query the site for additional information.

All personnel involved in the adjudication process will remain blinded to treatment allocation throughout the trial.

8.0 STATISTICAL ANALYSIS PLAN

This section outlines the statistical analysis strategy and procedures for the study. If, after the study has begun, but prior to any unblinding, changes made to primary and/or key secondary hypotheses, or the statistical methods related to those hypotheses, then the protocol will be amended (consistent with ICH Guideline E-9). Changes to exploratory or other non-confirmatory analyses made after the protocol has been finalized, but prior to unblinding, will be documented in a supplemental statistical analysis plan (sSAP) and referenced in the clinical study report (CSR) for the study. Post hoc exploratory analyses will be clearly identified in the CSR.

8.1 Statistical Analysis Plan Summary

Key elements of the statistical analysis plan are summarized below; the comprehensive plan is provided in Sections 8.2-8.12.

**Study Design Overview**	A randomized, active comparator-controlled trial evaluating the safety of sugammadex when used to reverse neuromuscular blockade induced by either rocuronium or vecuronium in ASA Class 3 or 4 subjects.
**Treatment Assignment**	Subjects will be stratified by NMBA (rocuronium or vecuronium) and ASA Class. Subjects will be randomized to depth of NMB (moderate or deep) and reversal agent (sugammadex or neostigmine + glycopyrrolate) as follows:<br>Within the rocuronium stratum, the treatment groups are:<br>1) Moderate block and reversal with sugammadex 2 mg/kg; or<br>2) Moderate block and reversal with neostigmine (50 µg/kg up to 5 mg maximum dose) + glycopyrrolate (10 µg/kg up to 1 mg maximum dose); or<br>3) Deep block and reversal with sugammadex 4 mg/kg; or<br>4) Deep block and reversal with sugammadex 16 mg/kg<br>Within the vecuronium stratum, the treatment groups are:<br>1) Moderate block and reversal with sugammadex 2 mg/kg; or<br>2) Moderate block and reversal with neostigmine (50 µg/kg up to 5 mg maximum dose) + glycopyrrolate (10 µg/kg up to 1 mg maximum dose); or<br>3) Deep block and reversal with sugammadex 4 mg/kg;
**Analysis Populations**	Safety: All Subjects as Treated (ASaT)
**Primary Endpoint(s)**	1) The proportion of subjects with treatment emergent sinus bradycardia<br>2) The proportion of subjects with treatment emergent sinus tachycardia<br>3) The proportion of subjects with other treatment emergent cardiac arrhythmias
**Key Secondary Endpoints**	N/A
**Statistical Methods for Key Efficacy/Immunogenicity/ Pharmacokinetic Analyses**	As the trial is focused on the evaluation of safety, no statistical evaluation for efficacy will be performed.
**Statistical Methods for Key Safety Analyses**	A tiered approach will be used to safety analyses. Tier 1 parameters will be subject to inferential testing for statistical significance with 95% confidence intervals (CI) for between-group comparisons. Tier 2 parameters will be assessed via point estimates with 95% confidence intervals provided for between-group comparisons; only point estimates by treatment group are provided for Tier 3 safety parameters.
**Interim Analyses**	No interim analyses are planned for this study.
**Multiplicity**	No multiplicity adjustment is planned.

**Sample Size and Power**	The total planned sample size is approximately 331 subjects. Approximately 30% of the total number of randomized subjects will be enrolled in the vecuronium stratum. Therefore, approximately 100 subjects will be enrolled in the vecuronium stratum and randomized to the 3 treatment arms in a 2:1:2 ratio; thus, there would be approximately 40 subjects in each of the sugammadex arms and approximately 20 subjects in the neostigmine arm. Approximately 231 subjects will be enrolled in the rocuronium stratum and randomized to the 4 treatment arms in a 2:1:2:2 ratio; thus, approximately 66 subjects will be in each of the sugammadex arms (i.e., 2 mg/kg, 4 mg/kg and 16 mg/kg) and approximately 33 subjects will be in neostigmine arm.

8.2 Interim Analyses

No interim analyses are planned for this study.

8.3 Responsibility for Analyses/In-House Blinding

The statistical analysis of the data obtained from this study will be the responsibility of the Clinical Biostatistics department of the Sponsor.

This study will be conducted as a double-blind study under in-house blinding procedures. With the exception of designated individuals (i.e., unblinded CRAs), all Sponsor study team personnel will be blinded to study medication assignment. The official, final database will not be unblinded until medical/scientific review has been performed, protocol deviations have been identified, and data have been declared final and complete.

The Clinical Biostatistics department will generate the randomized allocation schedule(s) for study treatment assignment. Randomization will be implemented using an IVRS.

8.4 Hypotheses/Estimation

Objectives of the study are stated in Section 3.0.

8.5 Analysis Endpoints

Only safety endpoints will be evaluated in this study. For safety analyses other than the primary safety endpoints (which includes events up to 30 minutes post administration of study medication), the primary approach to the summary of AEs will include all events that occur up to 7 days post administration of study medication. A supplemental summary of all AEs occurring up to 14 days post administration of study medication will also be included.

8.5.1 Tier 1 Safety Endpoints

The primary endpoints, which are considered as Tier 1 safety endpoints, are:

- Proportion of subjects with treatment emergent sinus bradycardia defined as a heart rate <60 bpm that has also decreased more than 20% compared to the subject's baseline heart rate value, sustained for at least 1 minute after administration of study medication.
- Proportion of subjects with treatment emergent sinus tachycardia defined as a heart rate ≥100 bpm that has increased more than 20% compared to the subject's baseline heart rate value, sustained for at least 1 minute after administration of study medication.

- Proportion of subjects with other treatment emergent cardiac arrhythmias defined as new or worsening arrhythmias (e.g., atrial fibrillation, atrial tachycardia, ventricular fibrillation, or ventricular tachycardia), sustained for at least 1 minute after administration of study medication.

The baseline heart rate for the primary safety endpoints will be recorded approximately 5 minutes before administration of study medication (sugammadex or neostigmine) to determine events of treatment emergent sinus bradycardia, treatment emergent sinus tachycardia, or other treatment emergent cardiac arrhythmias.

8.5.2 Tier 2 Safety Endpoints

Some supportive safety endpoints considered as Tier 2 endpoints are listed below:

- Proportion of subjects with adjudicated hypersensitivity.
- Proportion of subjects with adjudicated anaphylaxis.
- Proportion of subjects with at least one clinically relevant sinus bradycardia event defined as any bradycardia event necessitating intervention, as determined by investigator judgment.
- Proportion of subjects with at least one clinically relevant sinus tachycardia event defined as any tachycardia event necessitating intervention, as determined by investigator judgment.
- Proportion of subjects with a least one other clinically relevant cardiac arrhythmia defined as any other cardiac arrhythmia event necessitating intervention, as determined by investigator judgment.

In addition, proportion of subjects with any AE, serious AE, drug-related AE, serious and drug-related AE, discontinuation due to AE and specific AEs, SOCs, or PDLCs (incidence≥4 of subjects in one of the treatment group) will be considered as Tier 2 safety endpoints.

8.6 Analysis Populations

The All Subjects as Treated (ASaT) population will be used for the analysis of safety data in this study. The ASaT population consists of all randomized subjects who received at least one dose of study treatment. Subjects will be included in the treatment group corresponding to the study treatment they actually received for the analysis of safety data using the ASaT population. For most subjects this will be the treatment group to which they are randomized. Subjects who take incorrect study treatment will be included in the treatment group corresponding to the study treatment actually received.

At least one laboratory or vital sign measurement obtained subsequent to at least one dose of study treatment is required for inclusion in the analysis of each specific parameter. To assess change from baseline, a baseline measurement is also required.

8.7 Statistical Methods

Statistical testing and inference for safety analyses are described in Section 8.7.1. Unless otherwise stated, all statistical tests will be conducted at the α=0.05 (2-sided) level.

8.7.1 Statistical Methods for Safety Analyses

All safety analyses will be based on the All Subjects as Treated population.

Safety and tolerability will be assessed by clinical review of all relevant parameters including AEs, laboratory tests, and vital signs.

The analysis of safety results will follow a tiered approach (Table 5). The tiers differ with respect to the analyses that will be performed. Safety parameters or adverse experiences of special interest that are identified *a priori* constitute "Tier 1" safety endpoints that will be subject to inferential testing for statistical significance with p-values and 95% confidence intervals provided for between-group comparisons. Other safety parameters will be considered Tier 2 or Tier 3. Tier 2 parameters will be assessed via point estimates with 95% confidence intervals provided for between-group comparisons; only point estimates by treatment group are provided for Tier 3 safety parameters. Between-group comparisons will be made for each dosing group of sugammadex and neostigmine pooled across NMBA and separately by NMBA.

The incidence of safety parameters will be summarized by treatment group and preferred term across all 4 dosing groups. The primary safety endpoints are considered Tier 1 events. Both Tier 1 and Tier 2 endpoints will be estimated with 95% confidence interval by treatment groups using Clopper-Pearson method [6]. P-values (Tier 1 only) and 95% confidence interval (Tier 1 and Tier 2) will be provided for between-treatment differences using the stratified Miettinen and Nurminen method [7] with NMBA and ASA class as stratification factors.

Adverse experiences (specific terms as well as System Organ Class terms) and predefined limits of change in laboratory and vital signs that are not pre-specified as Tier-1 endpoints will be classified as belonging to Tier 2 or Tier 3, based on the number of events observed. Membership in Tier 2 requires that at least 4 subjects in any treatment group exhibit the event; all other adverse experiences and predefined limits of change will belong to Tier 3.

The threshold of at least 4 events was chosen because the 95% confidence interval for the between-group difference in percent incidence will always include zero when treatment groups of equal size each have fewer than 4 events and thus would add little to the interpretation of potentially meaningful differences. Because many 95% confidence intervals may be provided without adjustment for multiplicity, the confidence intervals should be regarded as a helpful descriptive measure to be used in review, not a formal method for assessing the statistical significance of the between-group differences in adverse experiences and predefined limits of change.

Continuous measures such as changes from baseline in laboratory and vital signs that are not pre-specified as Tier-1 endpoints will be considered Tier 3 safety parameters. Summary statistics for baseline, on-treatment, and change from baseline values will be provided by treatment group in table format. In addition, blood pressure will be evaluated in participants with Tier 1 safety events.

Table 5 summarizes the safety tier and level of analysis for the safety endpoints.

Table 5 Analysis Strategy for Safety Parameters

Safety Tier	Safety Endpoint[a]	p-Value	95% CI for Treatment Comparison	Descriptive Statistics
Tier 1	Treatment emergent sinus bradycardia	X	X	X
	Treatment emergent sinus tachycardia	X	X	X
	Other treatment emergent cardiac arrhythmias	X	X	X
Tier 2	Adjudicated hypersensitivity		X	X
	Adjudicated anaphylaxis		X	X
	Clinically relevant sinus bradycardia		X	X
	Clinically relevant sinus tachycardia		X	X
	Other clinically relevant cardiac arrhythmia		X	X
	Any AE		X	X
	Any serious AE		X	X
	Any drug-related AE		X	X
	Any serious and drug-related AE		X	X
	Discontinuation due to AE		X	X
	Specific AEs, SOCs, or PDLCs[b] (incidence ≥ 4 of subjects in one of the treatment groups)		X	X
Tier 3	Specific AEs, SOCs or PDLCs[b] (incidence <4 of subjects in all of the treatment groups)			X
	Change from Baseline[c] Results (Labs, Vital Signs)			X

[a] Adverse experience references refer to both clinical and laboratory AEs.
[b] Includes only those endpoints not pre-specified as Tier 1 or not already pre-specified as Tier 2 endpoints.
[c] Baseline measurements are taken at Visit 1.
Abbreviations: CI = Confidence interval; SOC = System Organ Class; PDLC = Pre-defined limit of change; X = Results will be provided.

8.7.2 Demographic and Baseline Characteristics

The comparability of the treatment groups for each relevant characteristic will be assessed by the use of tables and/or graphs. No statistical hypothesis tests will be performed on these characteristics. The number and percentage of subjects screened, randomized, the primary reasons for screening failure, and the primary reason for discontinuation will be displayed. Demographic variables (e.g., age, gender, BMI, race, ethnicity), baseline characteristics, surgical procedures, primary and secondary diagnoses, and prior and concomitant therapies will be summarized by treatment either by descriptive statistics or categorical tables.

8.8 Multiplicity

This study is to evaluate the safety profile of treatment. P-values and 95% confidence intervals for the primary safety endpoints (e.g., incidence of treatment emergent sinus bradycardia, treatment emergent sinus tachycardia, or other treatment emergent cardiac arrhythmia) will be provided for clinical interpretation, but there will not be any formal hypothesis. Therefore, no corrections for multiplicity are planned for this study.

8.9 Sample Size and Power Calculations

This study will enroll approximately 331 subjects in total. Approximately 30% of the total number of randomized subjects (100 subjects) will be administered vecuronium with a maximum of 70% of the total number of randomized subjects (231 subjects) to be administered rocuronium. The following calculations are conducted assuming these to be the numbers of subjects in each stratum.

Assuming 100 subjects will be enrolled in the vecuronium stratum and randomized to the 3 treatment arms in a 2:1:2 ratio, there would be 40 subjects in each of the sugammadex arms and 20 subjects in the neostigmine arm. If no events are observed among the 40 subjects in a given sugammadex group, an upper bound of the 95% confidence interval for subjects with any treatment emergent arrhythmia is ~8.8% (1 in every 11 subjects) in a treatment group. Assuming 231 subjects will be enrolled in the rocuronium stratum and randomized to the 4 treatment arms in a 2:1:2:2 ratio, there would be 66 subjects in each of the sugammadex arms (i.e., 2 mg/kg, 4 mg/kg, and 16 mg/kg) and 33 subjects in the neostigmine arm. If no events are observed among the 66 subjects in a given sugammadex group, an upper bound of the 95% CI for subjects with any treatment emergent arrhythmia is ~5.0% (1 in every 20 subjects) in a treatment group.

The estimate of incidence and the upper bound of the 95% CI for the underlying percentage of subjects with any treatment emergent arrhythmia within the sugammadex group are provided in Table 6. These calculations are based on the exact binomial method proposed by Clopper and Pearson (1934) [6].

Table 6 Estimate of Incidence of Any Treatment Emergent Arrhythmia and 95% Upper Confidence Bound Based on Hypothetical Number of Subjects with Any Treatment Emergent Arrhythmia Among 40 and 66 Subjects in the Sugammadex Group

Number of Subjects in Sugammadex Group	Hypothetical Number of Subjects With Any Treatment Emergent Arrhythmia	Estimate of Incidence	95% Upper Confidence Bound[a]
40	1	2.5%	13.2%
	3	7.5%	20.4%
	5	12.5%	26.8%
	7	17.5%	32.8%
	10	25.0%	41.2%
66	1	1.5%	8.2%
	3	4.6%	12.7%
	5	7.6%	16.8%
	7	10.6%	20.6%
	10	15.2%	26.1%

[a] Based on the two-tailed exact CI of a binomial proportion (Clopper and Pearson, 1934).

Table 7 summarizes the percentage point differences between the sugammadex and neostigmine groups for a variety of hypothetical underlying incidences of any treatment emergent arrhythmia. These calculations assume 66 subjects in the sugammadex group and 33 subjects in the neostigmine group and are based on a 2-sided 5% alpha level. The calculations are based on an asymptotic method proposed by Farrington and Manning (1990) [8]; no multiplicity adjustments were made.

Table 7 Differences in Incidence in Any Treatment Emergent Arrhythmia between the Sugammadex and Neostigmine Groups (Assuming Two-Sided 5% alpha level with 66 Subjects in Sugammadex Group and 33 Subjects in Neostigmine Group)

Incidence of Adverse Event		Risk Difference	
Sugammadex (%)	Neostigmine (%)	Percentage Points	95% CI[a]
1.5%	3.0%	1.5	(-13.9%, 5.5%)
3.0%	9.1%	6.1	(-20.9%, 3.2%)

Incidences presented here are hypothetical and do not represent actual adverse experiences in either group.

[a] Based on an asymptotic method (Farrington and Manning (1990)).

8.10 Subgroup Analyses and Effect of Baseline Factors

To determine whether the treatment effect is consistent across various subgroups, the estimate of the between-group treatment effect (with a nominal 95% CI) for the primary safety endpoint will be estimated across NMBA within each category of each subgroup. In addition, the consistency of the treatment effect on the primary endpoints will be assessed descriptively via summary statistics by category of subgroups for classification variables listed below:

- Age category (18-64, 65-74, or >74 years)
- Sex (female, male)
- BMI (<30 or ≥30 - <40 kg/m^2)
- Race (white, other)
- Ethnicity (Hispanic or Latino, Not Hispanic or Latino)
- ASA class (3, 4)
- Region (United States, non-United States)

Subgroup analyses/summaries will only be performed for those classification variables with ≥10% participants in each subgroup.

8.11 Compliance (Medication Adherence)

Compliance with dosage (in mg/kg) will be assessed based on the actual dosage (mg) of study medication administered and the reported body weight, per dosing occasion. Any dosage that differs by more than 10% from the planned dosage will be listed. No statistical tests will be performed with respect to treatment compliance.

8.12 Extent of Exposure

The extent of exposure to study medication will be summarized in a table presenting per treatment group the number of subjects who were randomized and received the study medication (i.e., were treated). If information on actual dose of study medication is available, summary statistics will be provided on the actual dose of study medication received (mg/kg).

9.0 LABELING, PACKAGING, STORAGE AND RETURN OF CLINICAL SUPPLIES

9.1 Investigational Product

The investigator shall take responsibility for and shall take all steps to maintain appropriate records and ensure appropriate supply, storage, handling, distribution and usage of investigational product in accordance with the protocol and any applicable laws and regulations.

Clinical Supplies will be provided by the Sponsor as summarized in Table 8.

Clinical supplies will be packaged to support enrollment and replacement subjects as required. When a replacement subject is required, the Sponsor or designee needs to be contacted prior to dosing the replacement supplies.

Table 8 Product Descriptions

Product Name & Potency	Dosage Form	Source/Additional Information
Sugammadex 100 mg/ml	Injection	Provided centrally by the Sponsor
Neostigmine methylsulfate 0.5mg/ml	Injection	Provided centrally by the Sponsor
Glycopyrrolate 0.2mg/ml	Injection	Provided centrally by the Sponsor

Sugammadex, neostigmine, and glycopyrrolate will be supplied to OR staff in masked syringes for administration to study subjects.

9.2 Packaging and Labeling Information

Clinical supplies will be affixed with a clinical label in accordance with regulatory requirements.

Clinical sites will receive open label single and multiple dose vials and kits. Each kit will contain the sufficient number of vials or ampoules to support treatment. Clinical supplies will be masked by the pharmacist (or delegate) as needed.

9.3 Clinical Supplies Disclosure

The emergency unblinding call center will use the treatment/randomization schedule for the trial to unblind subjects and to unmask treatment identity. In the event that the emergency unblinding call center is not available for a given site in this trial, the central electronic treatment allocation/randomization system (IVRS/IWRS) should be used in order to unblind subjects and to unmask treatment/vaccine identity. The Sponsor will not provide random code/disclosure envelopes or lists with the clinical supplies.

Treatment identification information is to be unmasked ONLY if necessary for the welfare of the subject. Every effort should be made not to unblind the subject unless necessary.

In the event that unblinding has occurred, the circumstances around the unblinding (e.g., date, reason and person performing the unblinding) must be documented promptly, and the Sponsor Clinical Director notified as soon as possible. Only the principal investigator or delegate and the respective subject's code should be unblinded. Trial site personnel and Sponsor personnel directly associated with the conduct of the trial should not be unblinded to treatment assignment. Subjects whose treatment assignment has been unblinded (by the investigator, Merck subsidiary, or through the emergency unblinding call center) must be discontinued from study drug, but should continue to be monitored in the trial.

9.4 Storage and Handling Requirements

Clinical supplies must be stored in a secure, limited-access location under the storage conditions specified on the label.

Receipt and dispensing of trial medication must be recorded by an authorized person at the trial site.

Clinical supplies may not be used for any purpose other than that stated in the protocol.

9.5 Discard/Destruction/Returns and Reconciliation

The investigator is responsible for keeping accurate records of the clinical supplies received from the Sponsor or designee, the amount dispensed to and returned by the subjects and the amount remaining at the conclusion of the trial. For all trial sites, the local country Sponsor personnel or designee will provide appropriate documentation that must be completed for drug accountability and return, or local discard and destruction if appropriate. Where local discard and destruction is appropriate, the investigator is responsible for ensuring that a local discard/destruction procedure is documented.

9.6 Standard Policies

Trial site personnel will have access to a central electronic treatment allocation/randomization system (IVRS/IWRS system) to allocate subjects, to assign treatment to subjects and to manage the distribution of clinical supplies. Each person accessing the IVRS system must be assigned an individual unique PIN. They must use only their assigned PIN to access the system, and they must not share their assigned PIN with anyone.

10.0 ADMINISTRATIVE AND REGULATORY DETAILS

10.1 Confidentiality

10.1.1 Confidentiality of Data

By signing this protocol, the investigator affirms to the Sponsor that information furnished to the investigator by the Sponsor will be maintained in confidence, and such information will be divulged to the institutional review board, ethics review committee (IRB/ERC) or similar or expert committee; affiliated institution and employees, only under an appropriate understanding of confidentiality with such board or committee, affiliated institution and employees. Data generated by this trial will be considered confidential by the investigator, except to the extent that it is included in a publication as provided in the Publications section of this protocol.

10.1.2 Confidentiality of Subject Records

By signing this protocol, the investigator agrees that the Sponsor (or Sponsor representative), IRB/ERC, or regulatory authority representatives may consult and/or copy trial documents in order to verify worksheet/case report form data. By signing the consent form, the subject agrees to this process. If trial documents will be photocopied during the process of verifying worksheet/case report form information, the subject will be identified by unique code only; full names/initials will be masked prior to transmission to the Sponsor.

By signing this protocol, the investigator agrees to treat all subject data used and disclosed in connection with this trial in accordance with all applicable privacy laws, rules and regulations.

10.1.3 Confidentiality of Investigator Information

By signing this protocol, the investigator recognizes that certain personal identifying information with respect to the investigator, and all subinvestigators and trial site personnel, may be used and disclosed for trial management purposes, as part of a regulatory submissions, and as required by law. This information may include:

1. name, address, telephone number and e-mail address;
2. hospital or clinic address and telephone number;
3. curriculum vitae or other summary of qualifications and credentials; and
4. other professional documentation.

Consistent with the purposes described above, this information may be transmitted to the Sponsor, and subsidiaries, affiliates and agents of the Sponsor, in your country and other countries, including countries that do not have laws protecting such information. Additionally, the investigator's name and business contact information may be included when reporting certain serious adverse events to regulatory authorities or to other investigators. By signing this protocol, the investigator expressly consents to these uses and disclosures.

If this is a multicenter trial, in order to facilitate contact between investigators, the Sponsor may share an investigator's name and contact information with other participating investigators upon request.

10.1.4 Confidentiality of IRB/IEC Information

The Sponsor is required to record the name and address of each IRB/IEC that reviews and approves this trial. The Sponsor is also required to document that each IRB/IEC meets regulatory and ICH GCP requirements by requesting and maintaining records of the names and qualifications of the IRB/IEC members and to make these records available for regulatory agency review upon request by those agencies.

10.2 Compliance with Financial Disclosure Requirements

Financial Disclosure requirements are outlined in the US Food and Drug Administration Regulations, Financial Disclosure by Clinical Investigators (21 CFR Part 54). It is the Sponsor's responsibility to determine, based on these regulations, whether a request for Financial Disclosure information is required. It is the investigator's/subinvestigator's responsibility to comply with any such request.

The investigator/subinvestigator(s) agree, if requested by the Sponsor in accordance with 21 CFR Part 54, to provide his/her financial interests in and/or arrangements with the Sponsor to allow for the submission of complete and accurate certification and disclosure statements. The investigator/subinvestigator(s) further agree to provide this information on a Certification/Disclosure Form, commonly known as a financial disclosure form, provided by the Sponsor. The investigator/subinvestigator(s) also consent to the transmission of this information to the Sponsor in the United States for these purposes. This may involve the transmission of information to countries that do not have laws protecting personal data.

10.3 Compliance with Law, Audit and Debarment

By signing this protocol, the investigator agrees to conduct the trial in an efficient and diligent manner and in conformance with this protocol; generally accepted standards of Good Clinical Practice (e.g., International Conference on Harmonization of Technical Requirements for Registration of Pharmaceuticals for Human Use Good Clinical Practice: Consolidated Guideline and other generally accepted standards of good clinical practice); and all applicable federal, state and local laws, rules and regulations relating to the conduct of the clinical trial.

The Code of Conduct, a collection of goals and considerations that govern the ethical and scientific conduct of clinical investigations sponsored by Merck, is provided in Section 12.1 - Merck Code of Conduct for Clinical Trials.

The investigator also agrees to allow monitoring, audits, IRB/ERC review and regulatory authority inspection of trial-related documents and procedures and provide for direct access to all trial-related source data and documents.

The investigator agrees not to seek reimbursement from subjects, their insurance providers or from government programs for procedures included as part of the trial reimbursed to the investigator by the Sponsor.

The investigator shall prepare and maintain complete and accurate trial documentation in compliance with Good Clinical Practice standards and applicable federal, state and local laws, rules and regulations; and, for each subject participating in the trial, provide all data, and, upon completion or termination of the clinical trial, submit any other reports to the Sponsor as required by this protocol or as otherwise required pursuant to any agreement with the Sponsor.

Trial documentation will be promptly and fully disclosed to the Sponsor by the investigator upon request and also shall be made available at the trial site upon request for inspection, copying, review and audit at reasonable times by representatives of the Sponsor or any regulatory authorities. The investigator agrees to promptly take any reasonable steps that are requested by the Sponsor as a result of an audit to cure deficiencies in the trial documentation and worksheets/case report forms.

The investigator must maintain copies of all documentation and records relating to the conduct of the trial in compliance with all applicable legal and regulatory requirements. This documentation includes, but is not limited to, the protocol, worksheets/case report forms, advertising for subject participation, adverse event reports, subject source data, correspondence with regulatory authorities and IRBs/ERCs, consent forms, investigator's curricula vitae, monitor visit logs, laboratory reference ranges, laboratory certification or quality control procedures and laboratory director curriculum vitae. By signing this protocol, the investigator agrees that documentation shall be retained until at least 2 years after the last approval of a marketing application in an ICH region or until there are no pending or contemplated marketing applications in an ICH region or until at least 2 years have elapsed since the formal discontinuation of clinical development of the investigational product. Because the clinical development and marketing application process is variable, it is anticipated that the retention period can be up to 15 years or longer after protocol database lock. The Sponsor will determine the minimum retention period and notify the investigator

when documents may be destroyed. The Sponsor will determine the minimum retention period and upon request, will provide guidance to the investigator when documents no longer need to be retained. The sponsor also recognizes that documents may need to be retained for a longer period if required by local regulatory requirements. All trial documents shall be made available if required by relevant regulatory authorities. The investigator must consult with and obtain written approval by the Sponsor prior to destroying trial and/or subject files.

ICH Good Clinical Practice guidelines recommend that the investigator inform the subject's primary physician about the subject's participation in the trial if the subject has a primary physician and if the subject agrees to the primary physician being informed.

The investigator will promptly inform the Sponsor of any regulatory authority inspection conducted for this trial.

Persons debarred from conducting or working on clinical trials by any court or regulatory authority will not be allowed to conduct or work on this Sponsor's trials. The investigator will immediately disclose in writing to the Sponsor if any person who is involved in conducting the trial is debarred or if any proceeding for debarment is pending or, to the best of the investigator's knowledge, threatened.

In the event the Sponsor prematurely terminates a particular trial site, the Sponsor will promptly notify that trial site's IRB/IEC.

According to European legislation, a Sponsor must designate an overall coordinating investigator for a multi-center trial (including multinational). When more than one trial site is open in an EU country, Merck, as the Sponsor, will designate, per country, a national principal coordinator (Protocol CI), responsible for coordinating the work of the principal investigators at the different trial sites in that Member State, according to national regulations. For a single-center trial, the Protocol CI is the principal investigator. In addition, the Sponsor must designate a principal or coordinating investigator to review the trial report that summarizes the trial results and confirm that, to the best of his/her knowledge, the report accurately describes the conduct and results of the trial [Clinical Study Report (CSR) CI]. The Sponsor may consider one or more factors in the selection of the individual to serve as the Protocol CI and or CSR CI (e.g., availability of the CI during the anticipated review process, thorough understanding of clinical trial methods, appropriate enrollment of subject cohort, timely achievement of trial milestones). The Protocol CI must be a participating trial investigator.

10.4 Compliance with Trial Registration and Results Posting Requirements

Under the terms of the Food and Drug Administration Amendments Act (FDAAA) of 2007 and the European Medicines Agency (EMA) clinical trial Directive 2001/20/EC, the Sponsor of the trial is solely responsible for determining whether the trial and its results are subject to the requirements for submission to http://www.clinicaltrials.gov, www.clinicaltrialsregister.eu or other local registries. Merck, as Sponsor of this trial, will review this protocol and submit the information necessary to fulfill these requirements. Merck entries are not limited to FDAAA or the EMA clinical trial directive mandated trials. Information posted will allow subjects to identify potentially appropriate trials for their disease conditions and pursue participation by calling a central contact number for further information on appropriate trial locations and trial site contact information.

By signing this protocol, the investigator acknowledges that the statutory obligations under FDAAA, the EMA clinical trials directive or other locally mandated registries are that of the Sponsor and agrees not to submit any information about this trial or its results to those registries.

10.5 Quality Management System

By signing this protocol, the Sponsor agrees to be responsible for implementing and maintaining a quality management system with written development procedures and functional area standard operating procedures (SOPs) to ensure that trials are conducted and data are generated, documented, and reported in compliance with the protocol, accepted standards of Good Clinical Practice, and all applicable federal, state, and local laws, rules and regulations relating to the conduct of the clinical trial.

10.6 Data Management

The investigator or qualified designee is responsible for recording and verifying the accuracy of subject data. By signing this protocol, the investigator acknowledges that his/her electronic signature is the legally binding equivalent of a written signature. By entering his/her electronic signature, the investigator confirms that all recorded data have been verified as accurate.

Detailed information regarding Data Management procedures for this protocol will be provided separately.

10.7 Publications

This trial is intended for publication, even if terminated prematurely. Publication may include any or all of the following: posting of a synopsis online, abstract and/or presentation at a scientific conference, or publication of a full manuscript. The Sponsor will work with the authors to submit a manuscript describing trial results within 12 months after the last data become available, which may take up to several months after the last subject visit in some cases such as vaccine trials. However, manuscript submission timelines may be extended on OTC trials. For trials intended for pediatric-related regulatory filings, the investigator agrees to delay publication of the trial results until the Sponsor notifies the investigator that all relevant regulatory authority decisions on the trial drug have been made with regard to pediatric-related regulatory filings. Merck will post a synopsis of trial results for approved products on www.clinicaltrials.gov by 12 months after the last subject's last visit for the primary outcome, 12 months after the decision to discontinue development, or product marketing (dispensed, administered, delivered or promoted), whichever is later.

These timelines may be extended for products that are not yet marketed, if additional time is needed for analysis, to protect intellectual property, or to comply with confidentiality agreements with other parties. Authors of the primary results manuscript will be provided the complete results from the Clinical Study Report, subject to the confidentiality agreement. When a manuscript is submitted to a biomedical journal, the Sponsor's policy is to also include the protocol and statistical analysis plan to facilitate the peer and editorial review of the manuscript. If the manuscript is subsequently accepted for publication, the Sponsor will allow the journal, if it so desires, to post on its website the key sections of the protocol that are relevant to evaluating the trial, specifically those sections describing the trial objectives

and hypotheses, the subject inclusion and exclusion criteria, the trial design and procedures, the efficacy and safety measures, the statistical analysis plan, and any amendments relating to those sections. The Sponsor reserves the right to redact proprietary information.

For multicenter trials, subsequent to the multicenter publication (or after public disclosure of the results online at www.clinicaltrials.gov if a multicenter manuscript is not planned), an investigator and his/her colleagues may publish their data independently. In most cases, publication of individual trial site data does not add value to complete multicenter results, due to statistical concerns. In rare cases, publication of single trial site data prior to the main paper may be of value. Limitations of single trial site observations in a multicenter trial should always be described in such a manuscript.

Authorship credit should be based on 1) substantial contributions to conception and design, or acquisition of data, or analysis and interpretation of data; 2) drafting the article or revising it critically for important intellectual content; and 3) final approval of the version to be published. Authors must meet conditions 1, 2 and 3. Significant contributions to trial execution may also be taken into account to determine authorship, provided that contributions have also been made to all three of the preceding authorship criteria. Although publication planning may begin before conducting the trial, final decisions on authorship and the order of authors' names will be made based on participation and actual contributions to the trial and writing, as discussed above. The first author is responsible for defending the integrity of the data, method(s) of data analysis and the scientific content of the manuscript.

The Sponsor must have the opportunity to review all proposed abstracts, manuscripts or presentations regarding this trial 45 days prior to submission for publication/presentation. Any information identified by the Sponsor as confidential must be deleted prior to submission; this confidentiality does not include efficacy and safety results. Sponsor review can be expedited to meet publication timelines.

11.0 LIST OF REFERENCES

[1] Fasting S, Gisvold SE. Serious intraoperative problems - a five-year review of 83,844 anesthetics. Can J Anesth 2002;49(6):545-53.

[2] Sprung J, Flick RP, Gleich SJ, Weingarten TN. Perioperative cardiac arrests. Signa Vitae 2008;3(2):8-12.

[3] Sprung J, Warner ME, Contreras MG, Schroeder DR, Beighley CM, Wilson GA, et al. Predictors of Survival following Cardiac Arrest in Patients Undergoing Noncardiac Surgery: A Study of 518,294 Patients at a Tertiary Referral Center. Anesthesiology 2003;99(2):259-69.

[4] Sampson HA, Munoz-Furlong A, Campbell RL, Adkinson J, Bock SA, Branum A, et al. Second symposium on the definition and management of anaphylaxis: Summary report: Second National Institute of Allergy and Infectious Disease/Food Allergy and Anaphylaxis Network symposium. J Allergy Clin Immunol 2006;117(2):391-7.

[5] American Society of Anesthesiologists. ASA physical status classification system [Internet]. Schaumburg, IL: ASA; 2017. Available from. https://www.asahq.org/resources/clinical-information/asa-physical-status-classification-system.

[6] Clopper CJ, Pearson ES. The use of confidence or Fiducial limits illustrated in the case of the binomial. Biometrika 1934;26(4):404-13.

[7] Miettinen O, Nurminen M. Comparative analysis of two rates. Stat Med 1985;4:213-26.

[8] Farrington CP, Manning G. Test statistics and sample size formulae for comparative binomial trials with null hypothesis of non-zero risk difference or non-unity relative risk. Stat Med 1990;9:1447-54.

12.0 APPENDICES

12.1 Merck Code of Conduct for Clinical Trials

Merck*
Code of Conduct for Clinical Trials

I. Introduction

A. Purpose

Merck, through its subsidiaries, conducts clinical trials worldwide to evaluate the safety and effectiveness of our products. As such, we are committed to designing, implementing, conducting, analyzing and reporting these trials in compliance with the highest ethical and scientific standards. Protection of subject safety is the overriding concern in the design of clinical trials. In all cases, Merck clinical trials will be conducted in compliance with local and/or national regulations and in accordance with the ethical principles that have their origin in the Declaration of Helsinki.

B. Scope

Such standards shall be endorsed for all clinical interventional investigations sponsored by Merck irrespective of the party (parties) employed for their execution (e.g., contract research organizations, collaborative research efforts). This Code is not intended to apply to trials which are observational in nature, or which are retrospective. Further, this Code does not apply to investigator-initiated trials which are not under the control of Merck.

II. Scientific Issues

A. Trial Conduct

1. Trial Design

Except for pilot or estimation trials, clinical trial protocols will be hypothesis-driven to assess safety, efficacy and/or pharmacokinetic or pharmacodynamic indices of Merck or comparator products. Alternatively, Merck may conduct outcomes research trials, trials to assess or validate various endpoint measures, or trials to determine subject preferences, etc.

The design (i.e., subject population, duration, statistical power) must be adequate to address the specific purpose of the trial. Research subjects must meet protocol entry criteria to be enrolled in the trial.

2. Site Selection

Merck selects investigative sites based on medical expertise, access to appropriate subjects, adequacy of facilities and staff, previous performance in Merck trials, as well as budgetary considerations. Prior to trial initiation, sites are evaluated by Merck personnel to assess the ability to successfully conduct the trial.

3. Site Monitoring/Scientific Integrity

Trial sites are monitored to assess compliance with the trial protocol and general principles of Good Clinical Practice. Merck reviews clinical data for accuracy, completeness and consistency. Data are verified versus source documentation according to standard operating procedures. Per Merck policies and procedures, if fraud, misconduct or serious GCP-non-Compliance are suspected, the issues are promptly investigated. When necessary, the clinical site will be closed, the responsible regulatory authorities and ethics review committees notified and data disclosed accordingly.

B. Publication and Authorship

To the extent scientifically appropriate, Merck seeks to publish the results of trials it conducts. Some early phase or pilot trials are intended to be hypothesis-generating rather than hypothesis testing. In such cases, publication of results may not be appropriate since the trial may be underpowered and the analyses complicated by statistical issues of multiplicity.

Merck's policy on authorship is consistent with the requirements outlined in the ICH-Good Clinical Practice guidelines. In summary, authorship should reflect significant contribution to the design and conduct of the trial, performance or interpretation of the analysis, and/or writing of the manuscript. All named authors must be able to defend the trial results and conclusions. Merck funding of a trial will be acknowledged in publications.

III. Subject Protection

A. IRB/ERC review

All clinical trials will be reviewed and approved by an independent IRB/ERC before being initiated at each site. Significant changes or revisions to the protocol will be approved by the IRB/ERC prior to implementation, except that changes required urgently to protect subject safety and well-being may be enacted in anticipation of IRB/ERC approval. For each site, the IRB/ERC and Merck will approve the subject informed consent form.

B. Safety

The guiding principle in decision-making in clinical trials is that subject welfare is of primary importance. Potential subjects will be informed of the risks and benefits of, as well as alternatives to, trial participation. At a minimum, trial designs will take into account the local standard of care. Subjects are never denied access to appropriate medical care based on participation in a Merck clinical trial.

All participation in Merck clinical trials is voluntary. Subjects are enrolled only after providing informed consent for participation. Subjects may withdraw from a Merck trial at any time, without any influence on their access to, or receipt of, medical care that may otherwise be available to them.

C. Confidentiality

Merck is committed to safeguarding subject confidentiality, to the greatest extent possible. Unless required by law, only the investigator, sponsor (or representative) and/or regulatory authorities will have access to confidential medical records that might identify the research subject by name.

D. Genomic Research

Genomic Research will only be conducted in accordance with informed consent and/or as specifically authorized by an Ethics Committee.

IV. Financial Considerations

A. Payments to Investigators

Clinical trials are time- and labor-intensive. It is Merck's policy to compensate investigators (or the sponsoring institution) in a fair manner for the work performed in support of Merck trials. Merck does not pay incentives to enroll subjects in its trials. However, when enrollment is particularly challenging, additional payments may be made to compensate for the time spent in extra recruiting efforts.

Merck does not pay for subject referrals. However, Merck may compensate referring physicians for time spent on chart review to identify potentially eligible subjects.

B. Clinical Research Funding

Informed consent forms will disclose that the trial is sponsored by Merck, and that the investigator or sponsoring institution is being paid or provided a grant for performing the trial. However, the local IRB/ERC may wish to alter the wording of the disclosure statement to be consistent with financial practices at that institution. As noted above, publications resulting from Merck trials will indicate Merck as a source of funding.

C. Funding for Travel and Other Requests

Funding of travel by investigators and support staff (e.g., to scientific meetings, investigator meetings, etc.) will be consistent with local guidelines and practices including, in the U.S., those established by the American Medical Association (AMA).

V. Investigator Commitment

Investigators will be expected to review Merck's Code of Conduct as an appendix to the trial protocol, and in signing the protocol, agree to support these ethical and scientific standards.

* In this document, "Merck" refers to Merck Sharp & Dohme Corp. and Schering Corporation, each of which is a subsidiary of Merck & Co., Inc. Merck is known as MSD outside of the United States and Canada. As warranted by context, Merck also includes affiliates and subsidiaries of Merck & Co., Inc."

12.2 Collection and Management of Specimens for Future Biomedical Research

1. Definitions

a. Biomarker: A biological molecule found in blood, other body fluids, or tissues that is a sign of a normal or abnormal process or of a condition or disease. A biomarker may be used to see how well the body responds to a treatment for a disease or condition.[1]

b. Pharmacogenomics: The investigation of variations of DNA and RNA characteristics as related to drug/vaccine response.[2]

c. Pharmacogenetics: A subset of pharmacogenomics, pharmacogenetics is the influence of variations in DNA sequence on drug/vaccine response.[2]

d. DNA: Deoxyribonucleic acid.

e. RNA: Ribonucleic acid.

2. Scope of Future Biomedical Research

The specimens consented and/or collected in this trial as outlined in Section 7.1.3.3 – Future Biomedical Research Samples will be used in various experiments to understand:

- o The biology of how drugs/vaccines work
- o Biomarkers responsible for how a drug/vaccine enters and is removed by the body
- o Other pathways drugs/vaccines may interact with
- o The biology of disease

The specimen(s) may be used for future assay development and/or drug/vaccine development.

It is now well recognized that information obtained from studying and testing clinical specimens offers unique opportunities to enhance our understanding of how individuals respond to drugs/vaccines, enhance our understanding of human disease and ultimately improve public health through development of novel treatments targeted to populations with the greatest need. All specimens will be used by the Sponsor or those working for or with the Sponsor.

3. Summary of Procedures for Future Biomedical Research

a. Subjects for Enrollment

All subjects enrolled in the clinical trial will be considered for enrollment in the Future Biomedical Research sub-trial.

b. Informed Consent

Informed consent for specimens (i.e., DNA, RNA, protein, etc.) will be obtained during screening for protocol enrollment from all subjects or legal guardians, at a trial visit by the investigator or his or her designate. Informed consent for Future Biomedical Research should be presented to the subjects on the visit designated in the trial flow chart. If delayed, present consent at next possible Subject Visit. Consent forms signed by the subject will be kept at the clinical trial site under secure storage for regulatory reasons.

A template of each trial site's approved informed consent will be stored in the Sponsor's clinical document repository.

c. eCRF Documentation for Future Biomedical Research Specimens

Documentation of subject consent for Future Biomedical Research will be captured in the electronic Case Report Forms (eCRFs). Any specimens for which such an informed consent cannot be verified will be destroyed.

d. Future Biomedical Research Specimen(s)

Collection of specimens for Future Biomedical Research will be performed as outlined in the trial flow chart. In general, if additional blood specimens are being collected for Future Biomedical Research, these will usually be obtained at a time when the subject is having blood drawn for other trial purposes.

4. Confidential Subject Information for Future Biomedical Research

In order to optimize the research that can be conducted with Future Biomedical Research specimens, it is critical to link subject' clinical information with future test results. In fact little or no research can be conducted without connecting the clinical trial data to the specimen. The clinical data allow specific analyses to be conducted. Knowing subject characteristics like gender, age, medical history and treatment outcomes are critical to understanding clinical context of analytical results.

To maintain privacy of information collected from specimens obtained for Future Biomedical Research, the Sponsor has developed secure policies and procedures. All specimens will be single-coded per ICH E15 guidelines as described below.

At the clinical trial site, unique codes will be placed on the Future Biomedical Research specimens. This code is a random number which does not contain any personally identifying information embedded within it. The link (or key) between subject identifiers and this unique code will be held at the trial site. No personal identifiers will appear on the specimen tube.

5. Biorepository Specimen Usage

Specimens obtained for the Sponsor will be used for analyses using good scientific practices. Analyses utilizing the Future Biomedical Research specimens may be performed by the Sponsor, or an additional third party (e.g., a university investigator) designated by the Sponsor. The investigator conducting the analysis will follow the Sponsor's privacy and confidentiality requirements. Any contracted third party analyses will conform to the specific scope of analysis outlined in this sub-trial. Future Biomedical Research specimens remaining with the third party after specific analysis is performed will be reported to the Sponsor.

6. Withdrawal From Future Biomedical Research

Subjects may withdraw their consent for Future Biomedical Research and ask that their biospecimens not be used for Future Biomedical Research. Subjects may withdraw consent at any time by contacting the principal investigator for the main trial. If medical records for the main trial are still available, the investigator will contact the Sponsor using the designated mailbox (clinical.specimen.management@merck.com).

Subsequently, the subject's specimens will be flagged in the biorepository and restricted to main study use only. If specimens were collected from study participants specifically for Future Biomedical Research, these specimens will be removed from the biorepository and destroyed. Documentation will be sent to the investigator confirming withdrawal and/or destruction, if applicable. It is the responsibility of the investigator to inform the subject of completion of the withdrawal and/or destruction, if applicable. Any analyses in progress at the time of request for withdrawal/destruction or already performed prior to the request being received by the Sponsor will continue to be used as part of the overall research trial data and results. No new analyses would be generated after the request is received.

In the event that the medical records for the main trial are no longer available (e.g., if the investigator is no longer required by regulatory authorities to retain the main trial records) or the specimens have been completely anonymized, there will no longer be a link between the subject's personal information and their specimens. In this situation, the request for withdrawal of consent and/or destruction cannot be processed.

7. Retention of Specimens

Future Biomedical Research specimens will be stored in the biorepository for potential analysis for up to 20 years from the end of the main study. Specimens may be stored for longer if a regulatory or governmental authority has active questions that are being answered. In this special circumstance, specimens will be stored until these questions have been adequately addressed.

Specimens from the trial site will be shipped to a central laboratory and then shipped to the Sponsor-designated biorepository. If a central laboratory is not utilized in a particular trial, the trial site will ship directly to the Sponsor-designated biorepository. The specimens will be stored under strict supervision in a limited access facility which operates to assure the integrity of the specimens. Specimens will be destroyed according to Sponsor policies and procedures and this destruction will be documented in the biorepository database.

8. Data Security

Databases containing specimen information and test results are accessible only to the authorized Sponsor representatives and the designated trial administrator research personnel and/or collaborators. Database user authentication is highly secure, and is accomplished using network security policies and practices based on international standards to protect against unauthorized access.

9. Reporting of Future Biomedical Research Data to Subjects

No information obtained from exploratory laboratory studies will be reported to the subject, family, or physicians. Principle reasons not to inform or return results to the subject include: Lack of relevance to subject health, limitations of predictive capability, and concerns regarding misinterpretation.

If important research findings are discovered, the Sponsor may publish results, present results in national meetings, and make results accessible on a public website in order to rapidly report this information to doctors and subjects. Subjects will not be identified by

name in any published reports about this study or in any other scientific publication or presentation.

10. Future Biomedical Research Study Population

Every effort will be made to recruit all subjects diagnosed and treated on Sponsor clinical trials for Future Biomedical Research.

11. Risks Versus Benefits of Future Biomedical Research

For future biomedical research, risks to the subject have been minimized. Risks include those associated with venipuncture to obtain the whole blood specimen. This specimen will be obtained at the time of routine blood specimens drawn in the main trial.

The Sponsor has developed strict security, policies and procedures to address subject data privacy concerns. Data privacy risks are largely limited to rare situations involving possible breach of confidentiality. In this highly unlikely situation there is risk that the information, like all medical information, may be misused.

12. Questions

Any questions related to the future biomedical research should be e-mailed directly to clinical.specimen.management@merck.com.

13. References

1. National Cancer Institute: http://www.cancer.gov/dictionary/?searchTxt=biomarker
2. International Conference on Harmonization: DEFINITIONS FOR GENOMIC BIOMARKERS, PHARMACOGENOMICS, PHARMACOGENETICS, GENOMIC DATA AND SAMPLE CODING CATEGORIES - E15; http://www.ich.org/products/guidelines/efficacy/efficacy-single/article/definitions-for-genomic-biomarkers-pharmacogenomics-pharmacogenetics-genomic-data-and-sample-cod.html
3. Industry Pharmacogenomics Working Group. Understanding the Intent, Scope and Public Health Benefits of Exploratory Biomarker Research: A Guide for IRBs/IECs and Investigational Site Staff. Available at http://i-pwg.org/
4. Industry Pharmacogenomics Working Group. Pharmacogenomics Informational Brochure for IRBs/IECs and Investigational Site Staff. Available at http://i-pwg.org/

12.3 Approximate Blood/Tissue Volumes Drawn/Collected by Trial Visit and by Sample Types

Trial Visit	Screening Visit 1	Peri-anesthetic visit Visit 2	Post anesthetic visit Visit 3
**Blood Parameter**	**Approximate Blood Volume (mL)**		
Hematology	0	2	2
Serum Chemistry	0	5	5
Serum β-Human Chorionic Gonadotropin (β-hCG)[a]	3.5		
Blood (DNA) for Future Biomedical Research	0	8.5	
Expected Total (mL)	3.5	15.5	7

a. For females of child bearing potential only. Sample taken only if the urine pregnancy test is positive at Visit 1 or if performed locally as part of standard of care.

12.4 List of Abbreviations and Definitions of Terms

Term	Definition
AE	Adverse event
ASA	American Society of Anesthesiologists
ASaT	All subjects as treated
BMI	Body mass index
BSA	Blinded Safety Assessor
CI	Confidence interval
CSR	Clinical study report
ECG	Electrocardiogram
ECI	Event of clinical interest
EDC	Electronic data capture
hCG	Human chorionic gonadotropin
ICH	International Conference on Harmonisation
IEC	Independent Ethics Committee
IRB	Institutional Review Board
IV	Intravenous
IVRS	Interactive Voice Response System
NMB<br>NMBA	Neuromuscular blockade<br>Neuromuscular blocking agent
NMTM	Neuromuscular Transmission Monitoring
OR	Operating room
PNS	Peripheral Nerve Stimulator
PTC	Post-tetanic count
SAE	Serious adverse event
T_1, T_2, T_3, T_4	First (T_1),second (T_2), third (T_3), or fourth (T_4) twitch in response to TOF stimulation
T_4/T_1 ratio	TOF ratio: Ratio of the height of T_4 over the height of T_1 in the recording of the response to TOF stimulation. Ratio expressed in decimals (e.g., 0.7 or 0.8 or 0.9)
TOF	Train of four stimulation

13.0 SIGNATURES

13.1 Sponsor's Representative

TYPED NAME	
TITLE	
SIGNATURE	
DATE SIGNED	

13.2 Investigator

I agree to conduct this clinical trial in accordance with the design outlined in this protocol and to abide by all provisions of this protocol (including other manuals and documents referenced from this protocol). I agree to conduct the trial in accordance with generally accepted standards of Good Clinical Practice. I also agree to report all information or data in accordance with the protocol and, in particular, I agree to report any serious adverse events as defined in Section 7.0 – TRIAL PROCEDURES (Assessing and Recording Adverse Events). I also agree to handle all clinical supplies provided by the Sponsor and collect and handle all clinical specimens in accordance with the protocol. I understand that information that identifies me will be used and disclosed as described in the protocol, and that such information may be transferred to countries that do not have laws protecting such information. Since the information in this protocol and the referenced Investigator's Brochure is confidential, I understand that its disclosure to any third parties, other than those involved in approval, supervision, or conduct of the trial is prohibited. I will ensure that the necessary precautions are taken to protect such information from loss, inadvertent disclosure or access by third parties.

TYPED NAME	
TITLE	
SIGNATURE	
DATE SIGNED	
